# Deriving disability weights for the Netherlands: findings from the Dutch disability weights measurement study

**DOI:** 10.1186/s12963-024-00342-0

**Published:** 2024-10-07

**Authors:** Juanita A. Haagsma, Periklis Charalampous

**Affiliations:** Department of Public Health, Erasmus MC University Medical Center, Rotterdam, The Netherlands

**Keywords:** Disability weight, Disability-adjusted life years, Health preference, Disease burden

## Abstract

**Background:**

The aims of this study were to establish national disability weights based on the health state preferences of a Dutch general population sample, examine the relation between results and respondent’s characteristics, and compare disability weights with those estimated in the European disability weights study.

**Methods:**

In this cross-sectional study, a web-based survey was administered to a general population 18–75 years from the Netherlands. The survey included paired comparison questions. Paired comparison data were analysed using probit regression and located results onto the 0-to-1 disability weight scale using non-parametric regression. Bootstrapping was used to estimate 95% uncertainty intervals (95%UI). Spearman’s correlation was used to investigate the relation of probit regression coefficients between respondent’s characteristics.

**Results:**

3994 respondents completed the questionnaire. The disability weights ranged from 0.007 (95%UI: 0.003–0.012) for mild distance vision impairment to 0.741 (95% UI: 0.498–0.924) for intensive care unit admission. Spearman’s correlation of probit coefficients between sub-groups based on respondent’s characteristics were all above 0.95 (*p* < 0.001). Comparison of disability weights of 140 health states that were included in the Dutch and European disability weights study showed a high correlation (Spearman’s correlation: 0.942; *p* < 0.001); however, for 76 (54.3%) health states the point estimate of the Dutch disability weight fell outside of the 95%UI of the European disability weights.

**Conclusions:**

Respondent’s characteristics had no influence on health state valuations with the paired comparison. However, comparison of the Dutch disability weights to the European disability weights indicates that health state preferences of the general population of the Netherlands differ from those of other European countries.

**Supplementary Information:**

The online version contains supplementary material available at 10.1186/s12963-024-00342-0.

## Background

In the early 1990s, the burden of disease concept was introduced. The burden of disease quantifies health loss in a population and has proven indispensable for monitoring population health, identifying major risk factors of ill health and guiding policy decisions in the field of public health [1, 2].

A frequently used metric to capture the burden of disease is the disability-adjusted life year (DALY). The DALY is a health gap measure that summarizes health loss due to premature mortality, expressed in years of life lost (YLL), and health loss due to time spent living in a situation of non-optimal health, expressed in years lived with disability (YLD). Pivotal for the calculation of the YLD are disability weights, which reflect the relative severity of health consequences of a health state on a 0-to-1 scale and which are based on the health state valuations of a panel of judges, such as health experts or the general population [1–5].

The DALY has been used in large international studies, such as the Global Burden of Disease (GBD) study and the Global Health Estimates, and many national studies [6–9]. The Dutch National Institute for Public Health and the Environment (RIVM) was one of the first national health institutes to apply the DALY metric in a national foresight study [10, 11]. Rather than using the GBD 1990 disability weights, a new set of disability weights was established that could be applied in a national context and that was more refined with regards to disease stages and health state descriptions compared to the GBD 1990 disability weights [12]. The Dutch disability weights were determined using the same methodological approach that was used to establish the GBD 1990 disability weights. This approach consists of asking medical experts to evaluate health states with ranking and person trade-off techniques. Particularly the person trade-off is a complex task that has been criticized as having limited validity and reliability [13–15].

After publication of the GBD 1990 and 1997 Dutch disability weights study, views with regards to appropriate disability weight measurement methods changed. One of the most important changes was the shift from incorporating health preferences of medical experts to incorporating health preferences of members of the general population [16, 17]. One of the reasons for this shift is that burden of disease studies are used for priority setting in health and guiding health policy decisions. It is therefore important to incorporate general populations’ perceptions and health state preferences, rather than health experts’ perceptions and health state preferences.

Linked to this shift with regards to study population is the shift towards the use of less complex health state preference elicitation methods, as some members of a study population consisting of persons from the general population may have lower numeracy or literacy levels compared to health experts [16, 17]. This means that complex health state elicitation methods, such as the person trade-off, that were used in earlier disability weights measurement studies targeting health experts cannot be used in general population samples. A frequently used method that has shown to elicit high quality information on health state preferences of a representative sample of the general population is the paired comparison technique. The paired comparison technique is grounded in Thurstone’s Law of Comparative Judgment theoretical framework and it has adequate reliability and good validity [18–21].

Based on these insights, GBD researchers developed a new methodological approach to determine a set of global disability weights [22]. Subsequently, a slightly refined version of this methodological protocol was used to establish a European set of disability weights and several national sets of disability weights [23–26]. However, in the Netherlands, the set of disability weights used to quantify YLD in the Dutch national burden of disease studies has not been updated since 1997. This means that the disability weights used in Dutch national forecast studies do not represent the Dutch general populations’ perceptions and health state preferences are based on valuation techniques that have theoretical challenges.

The primary aim of this study was to obtain a set of national disability weights based on the health state preferences of a representative sample of members from the Netherlands. The secondary aims of this study were to examine the relation between results and socio-demographic and health characteristics such age, gender, highest attained level of education, and chronic disease status, as well as to compare the resulting disability weights with those estimated in the European disability weights measurement study.

## Methods

### Study design

This is a cross-sectional observational study. We administered a web-based survey to a cohort of members of the general population of the Netherlands. Data were collected between 1 and 26 June 2023. Ethical approval was obtained from the Erasmus MC Ethics Review Board (MEC-2023-0239).

### Panel participants and eligibility criteria

Participants were recruited by Flycatcher Internet Research, a market research agency. Flycatcher Internet Research invited members from existing panels consisting of members of the general population residing in the Netherlands to complete the online survey. Inclusion criteria were: member of existing market research agency panel, aged 18–75 years, and sufficient command of the Dutch language. Exclusion criteria were age less than 18 years or older than 75 years. Information on age, gender, highest attained level of education, and region of residence of the individual Internet panel members was already known. Based on this information, Dutch panelists were invited to fill out the questionnaire to ensure national representativeness across age, gender, and highest attained level of education.

### Health states lay descriptions

A total of 210 health states were evaluated, of which 156 health states were included in the GBD 2010, European, or Japanese disability weights measurement studies [22–24]; 47 were new; and seven were included for experimental purposes and were not part of the Dutch disability weights measurement study. For the health states originating from preceding disability weights measurement studies, we either used the same health state descriptions as included in those studies or we modified the health state descriptions based on the advice of disease experts. For the 47 new health states, new health state descriptions were developed under the guidance of medical expert(s), aligning with the design principles employed in the GBD 2013 disability weights study (i.e. brief lay descriptions of up to 70 words were constructed, using simple and non-clinical vocabulary explaining the main symptoms and functional limitations of each health state) [22, 27]. A complete listing of all health states, their origins and lay descriptions used in this study can be found in the Additional file 1 (page 3).

### Health state elicitation technique

The paired comparison asks the participant to consider two hypothetical individuals (person A *versus* person B) with different health states and to indicate which person they regarded as healthier than the other (Additional file 1, page 2). Each respondent performed 18 paired comparison tasks. The health states depicted in these paired comparison tasks were drawn randomly, using a computer-generated algorithm from all available possible comparisons. We repeated the same pair of health states in the 2nd and 15th paired comparison questions, with health states presented in the same order (2nd question) and reverse order (15th question). This allowed us to evaluate the internal consistency and test re-test reliability of paired comparison responses.

### Socio-demographic and health characteristics

The survey also included questions about socio-demographic characteristics (e.g. gender, age, highest level of education, and region of residence). The highest level of education achieved was categorized into three groups according to the International Standard Classification of Education (ISCED) 2011: ISCED 0–2 (“Low”), ISCED 3–4 (“Middle”), and ISCED 5–8 (“High”). This categorization is in line with Statistics Netherlands’ adoption of ISCED as the Dutch standard for measuring educational directions. Additionally, the survey included questions about health characteristics (e.g. chronic disease status). Chronic disease status was measured by the presence of up to 11 chronic conditions (i.e. asthma or chronic bronchitis, heart disease, stroke, diabetes, arthritis, severe back complaints, arthrosis, cancer, memory problems, depression or anxiety disorder, and/or other problems). The number of chronic diseases was categorized into three groups: “Zero”, “One”, “Two or more”.

The data capture system did not allow for missing values, meaning that participants were required to respond to all questions. It did not allow participants to adjust their responses (i.e. to go back in the questionnaire).

### Statistical analysis

All statistical analyses were performed with R (version 4.1.0) and SPSS (version 28.0.1).

#### Socio-demographic and health characteristics:

Descriptive analyses were performed for socio-demographic and health characteristics data.

#### Test re-test analysis:

Paired comparison responses on the deliberate repetition of the 2nd and 15th questions were examined in the form of a test re-test analysis. The probability of choosing the same health state if the two health states were presented in the same order (2nd question) was calculated as the total number of consistent instances divided by the total number of responses. Similarly, the probability of choosing the same health state if the two health states were presented in reverse order (15th question) was calculated as the total number of inconsistent instances dived by the total number of responses. Inter-rater reliability was measured by Cohen’s Kappa (κ) [28, 29]; it allows for the assessment of agreement beyond what would be expected by chance by considering both the observed agreement and the expected agreement that would occur by chance. It ranges from − 1 to + 1 where values ≤ 0.20 indicate slight or no agreement; 0.21–0.40 fair agreement; 0.41–0.60 moderate agreement; 0.61–0.80 substantial agreement; and 0.81-1.0 almost perfect agreement. The level of agreement was examined by educational groups and regions.

#### Paired comparison responses:

Paired comparison data were first analysed based on the choice probabilities over all possible health state pairs. Response probabilities were ordered and plotted in a heatmap matrix. Paired comparison data were then analysed using probit regression analysis. A binary response variable was coded as 1 if the first health state in a pair was chosen as the healthier one; as -1 if the second health state in a pair was chosen as the healthier; and as 0 for health states other than the pair being considered. Probit regression of paired comparison responses provides estimates that capture the relative differences in valuation of health states and are on an arbitrary scale rather than on the 0-to-1 disability weight scale. Thus, an additional analytic step to anchor the resulting estimates onto the 0-to-1 disability weight scale was performed.

#### Anchor results from probit regression analysis:

To predict the resulting estimates of the probit regression on the 0-to-1 disability weight scale, a non-parametric regression model (loess) of the probit regression coefficients against the logit-transformed disability weights from the GBD 2013 disability weights study was ran [27]. Then, a bootstrapping approach with 1000 replicate samples with means defined by the predicted probit coefficients and variance by the standard deviation of the predicted probit coefficients was ran to estimate 95% uncertainty intervals (95%UI). On each bootstrap sample, a non-parametric model of the logit-transformed disability weights from the GBD 2013 disability weights study against the regression coefficients was fitted. An inverse logistic transformation was then applied to the mean predicted disability weights in order to obtain the Dutch disability weights on the 0-to-1 disability weight scale. Finally, the 95%UI were obtained from the corresponding distribution of the sampled disability weights.

#### Relation between probit coefficients and socio-demographic and health characteristics:

The relation of probit regression coefficients between age, gender, highest attained level of education, and chronic disease status was evaluated using the Spearman’s correlation. A correlation matrix was generated based on the probit coefficients associated with the above socio-demographic and health characteristics.

#### Comparison of the resulting disability weights to the European disability weights:

To test for differences between the Dutch and European disability weights [23], the Spearman’s correlation and the Wilcoxon signed-rank test was used.

### Availability of code

The statistical code used to derive the Dutch disability weights is available on GitHub (https://github.com/periklisch/Dutch_Disability_Weights.git*)* and included in the Additional file 2.

## Results

### Study population

Table 1 shows the socio-demographic and health characteristics of the respondents, as compared to the whole population distribution in the Netherlands. A total of 3994 respondents completed the web-based questionnaire. Due to the participant recruitment and survey distribution methods, the response rate could not be calculated. The age-gender distribution of the Dutch disability weight cohort sample *versus* the national population can be found in the Additional file 1 (page 24). The age-gender-education distribution of the participants by regional level can also be found in the Additional file 1 (page 25).

**Table 1:** Socio-demographic and health characteristics (*n* = 3994)

		Dutch disability weights cohort		National population [30, 31]	*p*-value^*^
		Number (*n*)	Percentage (%)	Percentage (%)	
Gender	Male	1976	49.5	49.7	0.968
	Female	2011	50.4	50.2	
	Other	7	0.2		
Age group	18–34 yrs.	1013	25.4	21.9	0.001
	35–54 yrs.	1450	36.3	25.5	
	55–75 yrs.	1531	38.3	26.0	
Education level	Low	894	22.4	22.5	0.025
	Middle	1948	48.8	38.9	
	High	1152	28.8	38.6	
Chronic conditions	0	2158	54.0		
	1	1163	29.1		
	2 or more	673	16.9		
Region of residence	North Netherlands	473	11.8	9.9	0.389
	East Netherlands	845	21.2	21.1	
	South Netherlands	901	22.6	21.0	
	West Netherlands	1775	44.4	47.9	

*p-value: from Chi-square test

### Paired comparison

**Fig. 1:**
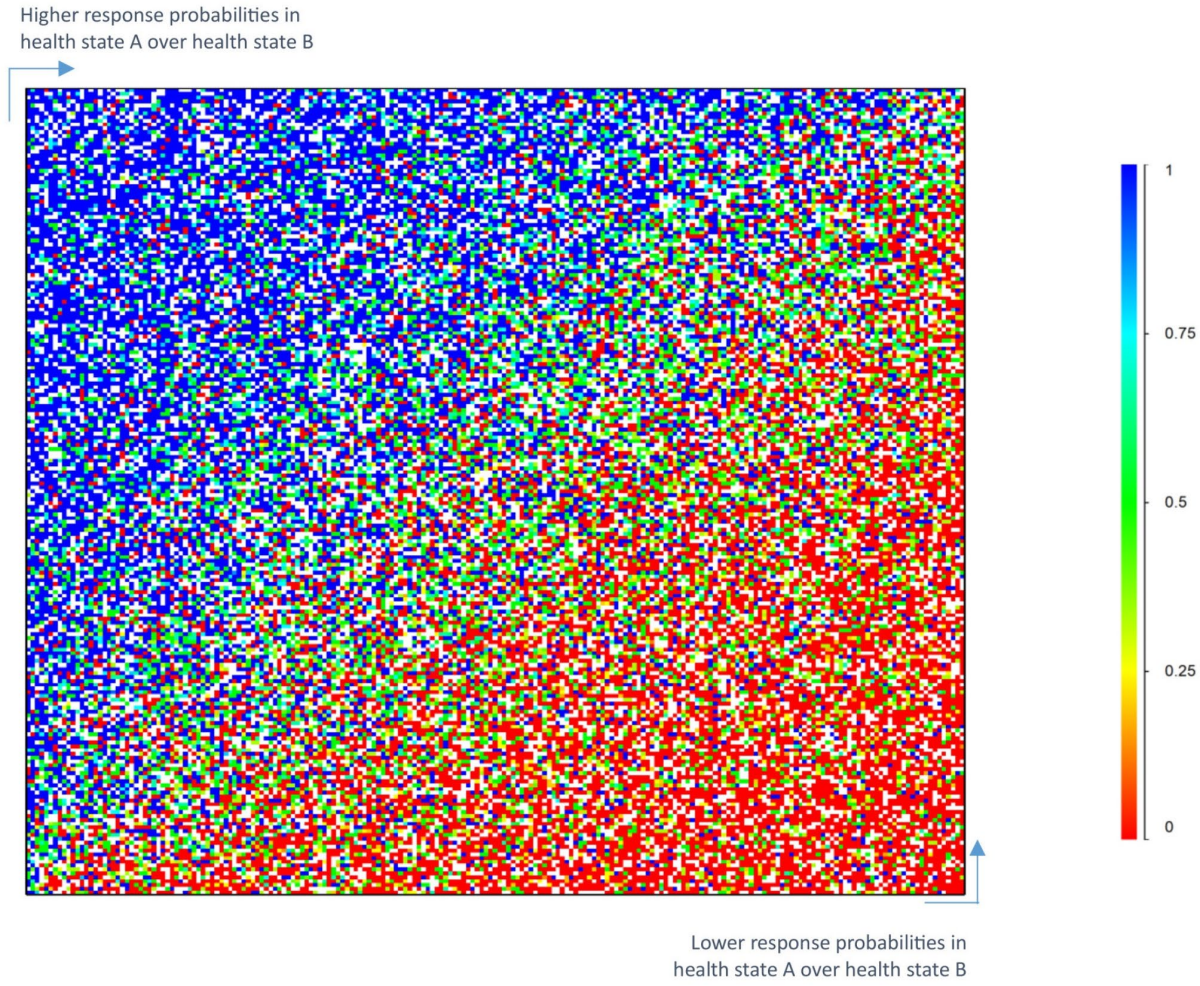
Response probabilities for paired comparisons

Figure 1 depicts a heatmap of the paired comparison response probabilities for the possible paired comparisons of 210 health states. Each cell in the heatmap represents the response probability for one pair of health states. Each colour represents the probability that the first health state in a pair comparison is chosen as the healthier outcome. Red cells correspond to probabilities less than 0.25; orange, yellow, and green cells correspond to probabilities between 0.25 and 0.75; and blue cells correspond to probabilities greater than 0.75. Please note that not all possible 210 × 210 pairs were evaluated by paired comparisons, which is indicated by some blanks in the figure. A completely smooth transition in colours from blue (upper left) to red (lower right) indicates a very small amount of measurement error and high internal consistency in paired comparison responses. Figure 1 does not show a completely smooth transition from blue to red.

Of the repeated paired comparison question 48.1% were presented in same order and 51.9% in reverse order. The probability of choosing the same health state was higher when they were presented in same order (0.741, κ = 0.482) compared to when they were presented in reverse order (0.727, κ=-0.453). Test re-test analysis of the paired comparison response probabilities by Dutch-pooled and Dutch-regions and educational level can be found in the Additional file 1 (page 26).

### Disability weights

Table 2 shows the estimated disability weights with 95%UI. The estimated disability weights ranged from 0.007 (95%UI: 0.003–0.012) for mild distance vision impairment to 0.741 (95% UI: 0.498–0.924) for intensive care unit admission. Of the 210 health states, 57 had a disability weight lower than 0.05. The lowest disability weights were associated with mild health states (e.g. mild neck pain: 0.055; 95%UI: 0.036–0.081), while the highest disability weights were associated with moderate (e.g. moderate neck pain: 0.146; 95%UI: 0.097–0.210) and severe (e.g. severe neck pain: 0.279; 95%UI: 0.191–0.377) health states.

**Table 2:** Estimated disability weights with uncertainty intervals

	Disability weight (95%UI)
**Infectious disease**	
Infectious disease: acute episode, mild	0.013 (0.007–0.020)
Infectious disease: acute episode, moderate	0.082 (0.056–0.115)
Infectious disease: acute episode, severe	0.213 (0.142–0.301)
Diarrhoea: with complications	0.436 (0.326–0.554)
Diarrhoea: without complications	0.145 (0.097–0.211)
HIV/AIDS: receiving antiretroviral treatment	0.106 (0.071–0.152)
AIDS: not receiving antiretroviral treatment	0.510 (0.404–0.622)
Tuberculosis: with HIV infection	0.391 (0.287–0.505)
Acute upper respiratory infections	0.015 (0.009–0.022)
**Cancer**	
Cancer, diagnosis and primary therapy	0.330 (0.241–0.430)
Cancer, metastatic	0.473 (0.362–0.595)
Mastectomy	0.064 (0.043–0.091)
Stoma	0.074 (0.049–0.104)
Terminal phase, with medication (for cancers, end-stage kidney/liver disease)	0.540 (0.432–0.663)
Terminal phase, without medication (for cancers, end-stage kidney/liver disease)	0.499 (0.389–0.617)
Cancer: residual stage, after treatment	0.181 (0.121–0.255)
**Cardiovascular and circulatory disease**	
Acute myocardial infarction: days 1–2	0.338 (0.238–0.444)
Cardiac conduction disorders and cardiac dysrhythmias	0.311 (0.221–0.414)
Heart failure: mild	0.087 (0.060–0.122)
Heart failure: moderate	0.139 (0.093–0.199)
Heart failure: severe	0.283 (0.196–0.380)
Stroke: long-term consequences, mild	0.013 (0.008–0.020)
Stroke: long-term consequences, moderate	0.103 (0.070–0.146)
Stroke: long-term consequences, severe	0.327 (0.233–0.434)
**Diabetes**,** digestive**,** and genitourinary disease**	
Diabetic foot	0.036 (0.023–0.052)
Diabetic neuropathy	0.206 (0.137–0.291)
Diabetes: without complications	0.061 (0.039–0.089)
Chronic kidney disease (stage III)	0.029 (0.018–0.044)
Chronic kidney disease (stage IV)	0.165 (0.112–0.238)
End-stage renal disease: on dialysis	0.556 (0.448–0.683)
Decompensated liver cirrhosis	0.324 (0.230–0.427)
Urinary incontinence	0.051 (0.034–0.075)
**Respiratory diseases**	
Asthma, controlled	0.017 (0.010–0.026)
Asthma, partially controlled	0.056 (0.036–0.082)
Asthma, uncontrolled	0.210 (0.142–0.293)
COPD and other chronic respiratory disease, mild	0.046 (0.030–0.068)
COPD and other chronic respiratory disease, moderate	0.330 (0.233–0.433)
COPD and other chronic respiratory disease, severe	0.405 (0.301–0.514)
**Neurological disorders**	
Dementia: mild	0.030 (0.019–0.044)
Dementia: moderate	0.293 (0.204–0.394)
Dementia: severe	0.266 (0.183–0.362)
Headache: migraine, mild	0.260 (0.178–0.357)
Headache: migraine, severe	0.410 (0.307–0.521)
Headache: tension-type	0.081 (0.055–0.113)
Multiple sclerosis: mild	0.145 (0.098–0.208)
Multiple sclerosis: moderate	0.286 (0.200-0.384)
Multiple sclerosis: severe	0.599 (0.476–0.739)
Idiopathic epilepsy: less severe (seizures < 12 per year)	0.344 (0.246–0.450)
Idiopathic epilepsy: severe (seizures ≥ 1 per month)	0.503 (0.389–0.630)
Parkinson’s disease: mild	0.026 (0.017–0.038)
Parkinson’s disease: moderate	0.343 (0.241–0.447)
Parkinson’s disease: severe	0.596 (0.477–0.728)
**Mental**,** behavioural**,** and substance use disorders**	
Alcohol use disorder: mild	0.287 (0.199–0.385)
Alcohol use disorder: severe	0.518 (0.408–0.643)
Drug dependence: mild	0.075 (0.050–0.107)
Drug dependence: moderate to severe	0.507 (0.399–0.627)
Anxiety disorders: mild	0.043 (0.028–0.062)
Anxiety disorders: moderate	0.151 (0.102–0.217)
Anxiety disorders: severe	0.503 (0.395–0.625)
Major depressive disorder: mild episode	0.153 (0.102–0.220)
Major depressive disorder: moderate episode	0.400 (0.295–0.508)
Major depressive disorder: severe episode	0.537 (0.426–0.669)
Burnout: minor complications	0.114 (0.077–0.165)
Burnout: major complications	0.277 (0.189–0.375)
Bipolar disorder: manic episode	0.373 (0.275–0.485)
Bipolar disorder: residual state	0.050 (0.032–0.073)
Schizophrenia: acute state	0.341 (0.249–0.442)
Schizophrenia: residual state	0.468 (0.362–0.591)
Anorexia nervosa	0.470 (0.360–0.598)
Bulimia nervosa	0.300 (0.208–0.399)
Binge eating disorder	0.214 (0.143–0.299)
Other specified feeding and eating disorder	0.508 (0.393–0.636)
Attention deficit hyperactivity disorder: mild	0.027 (0.017–0.042)
Attention deficit hyperactivity disorder: moderate	0.033 (0.021–0.050)
Attention deficit hyperactivity disorder: severe	0.073 (0.048–0.106)
Autism: moderate	0.045 (0.029–0.066)
Autism: severe	0.169 (0.110–0.250)
Intellectual disability: borderline (IQ 70/75–85/90)	0.020 (0.012–0.030)
Intellectual disability: mild (IQ 50/55–70)	0.041 (0.027–0.061)
Intellectual disability: moderate (IQ 35/40–50/55)	0.088 (0.061–0.125)
Intellectual disability: severe (IQ 20/25–35/40)	0.089 (0.060–0.125)
Intellectual disability: profound (IQ less than 20–25)	0.076 (0.050–0.108)
Personality disorders: mild	0.148 (0.098–0.211)
Personality disorders: moderate	0.252 (0.174–0.341)
Personality disorders: severe	0.497 (0.386–0.620)
**Hearing and vision loss**	
Hearing loss: mild	0.009 (0.005–0.015)
Hearing loss: moderate	0.018 (0.011–0.027)
Hearing loss: severe	0.175 (0.116–0.252)
Hearing loss: profound	0.197 (0.133–0.275)
Hearing loss: complete	0.232 (0.156–0.325)
Hearing loss: mild with ringing	0.019 (0.011–0.028)
Hearing loss: moderate with ringing	0.056 (0.036–0.082)
Hearing loss: severe with ringing	0.270 (0.184–0.365)
Hearing loss: profound with ringing	0.302 (0.206–0.403)
Hearing loss: complete with ringing	0.393 (0.288–0.503)
Unilateral hearing loss	0.012 (0.006–0.018)
Distance vision: mild impairment	0.007 (0.003–0.012)
Distance vision: moderate impairment	0.021 (0.013–0.031)
Distance vision: severe impairment	0.132 (0.090–0.187)
Distance vision: blindness	0.145 (0.097–0.212)
Distance vision: monocular impairment	0.018 (0.011–0.027)
Near vision impairment	0.008 (0.004–0.013)
**Musculoskeletal disorders**	
Low back pain: mild	0.028 (0.018–0.042)
Low back pain: moderate	0.073 (0.048–0.105)
Low back pain: severe (without leg pain)	0.276 (0.194–0.371)
Neck pain: mild	0.055 (0.036–0.081)
Neck pain: moderate	0.146 (0.097–0.210)
Neck pain: severe	0.279 (0.191–0.377)
Musculoskeletal problems, lower limbs: moderate	0.099 (0.069–0.137)
Musculoskeletal problems, lower limbs: severe	0.212 (0.143–0.297)
Musculoskeletal problems, upper limbs: moderate	0.116 (0.078–0.164)
Musculoskeletal problems: generalized, moderate	0.327 (0.233–0.432)
Musculoskeletal problems: generalized, severe	0.610 (0.487–0.745)
Gout: acute, mild	0.026 (0.016–0.039)
Gout: acute, moderate	0.051 (0.034–0.076)
Gout: acute, severe	0.327 (0.235–0.427)
**Injury**	
Amputation of finger(s), excluding thumb	0.019 (0.012–0.029)
Amputation of thumb: long term	0.027 (0.017–0.040)
Amputation of one upper limb: long term, with treatment	0.045 (0.029–0.066)
Amputation of one upper limb: long term, without treatment	0.096 (0.065–0.139)
Amputation of both upper limbs: long term, with treatment	0.110 (0.076–0.156)
Amputation of both upper limbs: long term, without treatment	0.237 (0.160–0.332)
Amputation of toe	0.018 (0.011–0.027)
Amputation of one lower limb: long term, with treatment	0.037 (0.024–0.055)
Amputation of one lower limb: long term, without treatment	0.219 (0.145–0.301)
Amputation of both lower limbs: long term, with treatment	0.093 (0.063–0.132)
Amputation of both lower limbs: long term, without treatment	0.525 (0.423–0.640)
Burns, < 20% total burned surface area without lower airway burns: short term, with or without treatment	0.042 (0.027–0.060)
Burns, < 20% total burned surface area or < 10% total burned surface area if head/neck or hands/wrist involved: long term, with or without treatment	0.024 (0.015–0.036)
Burns, ≥ 20% total burned surface area: short term, with or without treatment	0.192 (0.130–0.272)
Burns, ≥ 20% total burned surface area or ≥ 10% total burned surface area if head/neck or hands/wrist involved: long term, with treatment	0.147 (0.098–0.209)
Burns, ≥ 20% total burned surface area or ≥ 10% total burned surface area if head/neck or hands/wrist involved: long term, without treatment	0.419 (0.318–0.529)
Crush injury: short or long term, with or without treatment	0.088 (0.059–0.125)
Dislocation of hip: long term, with or without treatment	0.041 (0.027–0.061)
Dislocation of knee: long term, with or without treatment	0.048 (0.031–0.071)
Dislocation of shoulder: long term, with or without treatment	0.037 (0.025–0.054)
Other injuries of muscle and tendon (includes sprains, strains and dislocations other than shoulder, knee, hip)	0.016 (0.009–0.024)
Drowning and nonfatal submersion: short or long term, with or without treatment	0.234 (0.159–0.328)
Fracture of clavicle, scapula or humerus: short or long term, with or without treatment	0.026 (0.017–0.039)
Fracture of foot bones: short term, with or without treatment	0.014 (0.009–0.022)
Fracture of foot bones: long term, without treatment	0.037 (0.024–0.056)
Fracture of hand: short term, with or without treatment	0.017 (0.010–0.026)
Fracture of hand: long term, without treatment	0.019 (0.011–0.028)
Fracture of neck of femur: short term, with or without treatment	0.099 (0.068–0.140)
Fracture of neck of femur: long term, with treatment	0.041 (0.026–0.060)
Fracture other than femoral neck: short term, with or without treatment	0.016 (0.010–0.024)
Fracture other than femoral neck: long term, without treatment	0.040 (0.026–0.058)
Fracture of patella, tibia or fibula or ankle: short term, with or without treatment	0.030 (0.020–0.045)
Fracture of patella, tibia or fibula or ankle: long term, with or without treatment	0.050 (0.033–0.074)
Fracture of pelvis: short term	0.217 (0.144–0.304)
Fracture of pelvis: long term	0.186 (0.125–0.267)
Fracture of radius or ulna: short term, with or without treatment	0.029 (0.018–0.043)
Fracture of radius or ulna: long term, without treatment	0.044 (0.029–0.066)
Fracture of skull: short or long term, with or without treatment	0.092 (0.062–0.133)
Fracture of sternum and/or fracture of one or two ribs: short term, with or without treatment	0.055 (0.036–0.081)
Fracture of vertebral column: short or long term, with or without treatment	0.065 (0.042–0.094)
Fractures, treated: long term	0.009 (0.004–0.014)
Injured nerves: short term	0.102 (0.069–0.143)
Injured nerves: long term	0.280 (0.192–0.377)
Injury to eyes: short term	0.044 (0.029–0.064)
Concussion	0.091 (0.062–0.130)
Traumatic brain injury: severe, short term, with or without treatment	0.159 (0.106–0.224)
Traumatic brain injury, long-term consequences, minor, with or without treatment	0.093 (0.063–0.134)
Traumatic brain injury, long-term consequences, moderate, with or without treatment	0.174 (0.117–0.250)
Traumatic brain injury, long-term consequences, severe, with or without treatment	0.517 (0.410–0.635)
Open wound: short term, with or without treatment	0.008 (0.004–0.013)
Poisoning: short term with or without treatment	0.144 (0.096–0.205)
Severe chest injury: short term, with or without treatment	0.343 (0.248–0.448)
Severe chest injury: long term, with or without treatment	0.051 (0.034–0.074)
Spinal cord lesion below neck level: treated	0.299 (0.212–0.399)
Spinal cord lesion below neck level: untreated	0.589 (0.470–0.729)
Spinal cord lesion at neck level: treated	0.519 (0.410–0.651)
Spinal cord lesion at neck level: untreated	0.709 (0.514–0.887)
Injury to internal organs	0.471 (0.359–0.587)
Broken teeth	0.051 (0.033–0.075)
Broken nose	0.010 (0.005–0.016)
Broken cheekbone	0.030 (0.019–0.044)
**Other**	
Annoyance: moderate	0.048 (0.032–0.071)
Annoyance: severe	0.066 (0.043–0.096)
Cognitive impairments: mild	0.009 (0.004–0.015)
Cognitive impairments: moderate	0.088 (0.058–0.124)
Cognitive impairments: severe	0.161 (0.106–0.232)
Constitutional eczema: mild/moderate	0.019 (0.012–0.029)
Constitutional eczema: severe	0.167 (0.112–0.237)
Fatigue	0.065 (0.042–0.094)
Impaired self-care	0.051 (0.033–0.075)
Intensive care unit admission	0.741 (0.498–0.924)
Loneliness	0.028 (0.017–0.041)
Motor impairment: mild	0.010 (0.005–0.016)
Motor impairment: moderate	0.062 (0.041–0.088)
Motor impairment: severe	0.263 (0.182–0.360)
Motor and cognitive impairments: mild	0.031 (0.020–0.045)
Motor and cognitive impairments: moderate	0.116 (0.078–0.169)
Motor and cognitive impairments: severe	0.393 (0.291–0.502)
Sleep disturbance	0.058 (0.047–0.070)
Vertigo and balance disorder (Menière, labyrinthitis)	0.089 (0.059–0.129)
Generic, mild health problems	0.009 (0.004–0.015)
Generic, mild physical and mental health problems	0.024 (0.015–0.036)
Generic, mild physical and mental health problems and mild functional limitations	0.031 (0.020–0.045)
Generic, mild mental health problems and moderate physical health problems and functional limitations	0.073 (0.048–0.104)
Generic, moderate mental health problems and severe physical health problems and functional limitations	0.208 (0.135–0.293)
Generic, severe physical and mental health problems and severe functional limitations	0.242 (0.165–0.334)
Generic, extreme physical and mental health problems and extreme functional limitations	0.423 (0.320–0.538)

### Relation between probit coefficients and socio-demographic and health characteristics

**Fig. 2:**
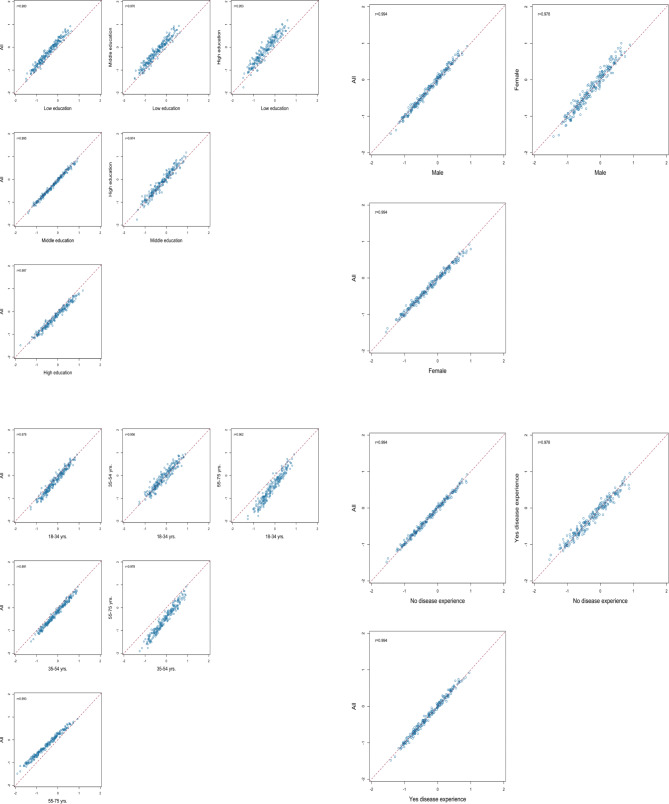
Correlation of probit coefficients by highest attained level of education, gender, age category, and chronic disease status in the Dutch disability weights cohort sample

Figure 2 illustrates the Spearman’s correlation of the probit coefficients between sub-groups based on education level, gender, age category, and chronic disease status. Spearman’s correlations were all above 0.95 (education level r_s_: 0.953–0.995; age categories r_s_: 0.956–0.978; gender r_s_: 0.978; and chronic disease status r_s_: 0.980, all *p* < 0.001), Additional file 1 (pages 27–29).

### Comparison to the European disability weights

Comparison of the disability weights of the 140 health states that were included in both the Dutch and European disability weights study showed a high correlation (Spearman’s correlation: 0.942; *p* < 0.001). The median disability weight of all 140 health states did not differ significantly between the Dutch and the European disability weight study (median of Dutch disability weights: 0.103, interquartile range (IQR) 0.041–0.291; median of European disability weights: 0.122, IQR 0.044–0.281); *p* = 0.972). However, for 76 (54.3%) of the 140 health states the point estimate of the Dutch disability weight fell outside of the 95%UI of the European disability weights. For 33 (23.6%) health states, the Dutch disability weights were lower than the lower bound. Approximately one in three of the neurological and injury health states was significantly lower than the European disability weights (Additional file 1, page 30). For 43 (30.7%) health states, the Dutch disability weights were higher than the higher bound of the 95%UI of the European disability weights. All of the diabetes, digestive, and genitourinary disease health states and four in five cardiovascular and circulatory diseases were significantly higher than the European disability weights (Additional file 1, page 30).

## Discussion

This study determined disability weights based on health preferences of a Dutch general population sample. The resulting disability weights ranged from 0.007 (95%UI: 0.003–0.012) for mild distance vision impairment to 0.741 (95% UI: 0.498–0.924) for intensive care unit admission. Diseases with multiple stages in terms of severity (e.g. traumatic brain injury and hearing loss) had a logical ranking with lowest disability weights attributed to mild stages and the highest disability weights to severe stages.

However, the ranking of certain health states with multiple severity stages seems counterintuitive. For example, the disability weight for profound intellectual disability (0.076, 95%UI: 0.050–0.108) was estimated to be lower than the disability weight for severe intellectual disability (0.089, 95%UI: 0.060–0.125) and moderate intellectual disability (0.088, 95%UI: 0.061–0.125). A possible explanation for this difference may be that a modified health state description for profound intellectual disability was used. This may elicit differences in disability weights, and it is likely that the major functional consequences and symptoms associated with moderate and severe *versus* profound intellectual disability were not fully understood by the Dutch general population. Another noteworthy observation was the difference between moderate (0.293, 95% 0.204–0.394) and severe (0.266, 95%UI: 0.183–0.362) dementia. It should be noted that a similar observation was identified in the European NOISE disability weights measurement study [26]. This underscores the importance of adapting brief lay descriptions for dementia in future disability weights measurement studies.

We found high correlations of the probit coefficients between sub-groups based on educational level, age category, gender, and region of residence. This indicates that socio-demographic characteristics had no influence on health state valuations with the paired comparison. These findings are in agreement with those of previous disability weight measurement studies [25, 32, 33].

Comparison of the Dutch disability weights to the European disability weights showed that for slightly more than half of the health states the value of the Dutch and European disability weights differed significantly. Notably many health states in disease categories cancer, neurological disorders, injury and other health states had a significantly lower disability weight compared to the European disability weights. On the other hand, many of the health states in disease categories diabetes, digestive, and genitourinary disease health states and cardiovascular and circulatory diseases and infectious disease had a significantly higher disability weight compared to the European disability weights [23]. These differences in disability weights between the Dutch and European studies may be due to differences in health state preferences across countries. The study population of the European disability weight study consisted of members of the general population of four countries, namely Hungary, Italy, the Netherlands and Sweden [23]. Comparison of the responses on the paired comparison task showed that there was more variation in health states valuation between countries than between other respondent characteristics [32]. Particularly the correlation of the probit coefficients between the Netherlands and Italy, Hungary and Sweden was lower compared to the correlations between Hungary, Italy and Sweden. This may indicate that health state preferences of the general population of the Netherlands differ from those of other European countries.

Another reason for differences in health state preferences between the Dutch and European study could be that health state preferences may have changed over time. The data of the European disability weights study were collected in 2013, ten years before data collection of the Dutch disability weight study. In the meantime, the COVID-19 pandemic occurred and in January 2023, 8.6 million COVID-19 cases had been recorded in the Netherlands (17.6 million inhabitants) [34]. Recent experience with a COVID-19 infection themselves or in a significant other may have had an effect on the respondent’s health state preferences. The relatively high percentage of infectious disease and respiratory disease health states with a significantly higher disability weight compared to the European disability weights may point in that direction. However, the link between experiencing COVID-19 infection and changes in health state preferences with regards to diabetes, digestive, and genitourinary disease and cardiovascular and circulatory diseases health states is less obvious. In addition, the findings of our study showed that having one or more chronic disease(s) had no influence on respondents’ health state valuations.

Comparison of the disability weights derived in this study to those of the 1997 Dutch disability weights study shows that the 1997 Dutch disability weights almost cover the full 0-to-1 disability weight scale and are on average higher [12]. The values of the 1997 Dutch disability weights range from 0 (gingivitis) to 0.93 (terminally ill), whereas the disability weight established this study range from 0.007 to 0.741. This can be explained by the differences in health state valuation techniques and statistical methods that were used to derive the disability weights in the two studies. As a result, the values of the disability weights of the two Dutch disability weight studies are incomparable and the disability weights cannot be used together in the same study [17].

A strength of our study is that the disability weights are tailored to the epidemiological data that are used to determine the burden of disease of diseases, injuries and risk factors in the Netherlands. This means that for several diseases disability weights were established on a more detailed level (e.g. multiple stages for asthma, diabetes, autism) compared to the GBD 2013 disability weights. Furthermore, data were collected via a web-based survey among a panel of respondents that were representative for the adult Dutch population in terms of age, gender, and educational level. Although collecting data via a web-based survey may affect data quality person [34], our study showed substantial test-retest reliability of the paired comparison responses, indicating high quality of responses.

A limitation of this study is that we used the GBD 2013 disability weights to anchor the Dutch disability weights, rather than population health equivalence questions as was done in GBD 2010 and 2013 study and the Chinese disability weight study [22, 25, 27]. As result, the disability weights established in this study are not based on trade-offs between non-fatal and fatal outcomes of the Dutch general population, but on the GBD study population, which covered many countries from different world regions [27]. However, previous studies have shown that the quality of population health equivalence data derived via a web-based survey among a sample of the general population is low and we therefore choice for this alternative anchoring strategy [23, 24].

In conclusion, we observed logical ranking of the disability weights that were established in this study, with lowest disability weights attributed to mild health states and the highest disability weights to severe health states. Socio-demographic and health characteristics had no influence on health state valuations with the paired comparison. However, comparison of the Dutch disability weights to the European disability weights indicates that health state preferences of the general population of the Netherlands differ from those of other European countries.

## Electronic supplementary material

Below is the link to the electronic supplementary material.

Supplementary Material 1

Supplementary Material 2

## Data Availability

The dataset supporting the conclusions of the current study is available for researchers who meet the criteria for access to data upon request which can be applied at the Data Access Committee of the Department of Public Health of the Erasmus MC under the MEC-2023-0239 reference number. The statistical code used to derive the Dutch disability weights is available on GitHub (https://github.com/periklisch/Dutch_Disability_Weights.git*)* and included in the Additional file 2.
