# Supplementary material for: Deriving disability weights for the Netherlands: findings from the Dutch disability weights measurement study
Source: Popul Health Metr. 2024 Oct 7;22:26. doi: 10.1186/s12963-024-00342-0 (PMC11457395; doi:10.1186/s12963-024-00342-0)
Supplement: Supplementary file 1 — Supplementary Material 1 [file 12963_2024_342_MOESM1_ESM.pdf]

Supplementary file to *“Deriving disability weights for the Netherlands: findings from the Dutch disability weights measurement study”*

The following supplementary file provides more detailed methodology and supplemental figures and results for *"Deriving disability weights for the Netherlands: findings from the Dutch disability weights measurement study”*

Table of contents

1. Statistical analysis: paired comparison
2. Supplemental tables and figures
2.1. Table 1: Brief lay descriptions for health states and their origins (in English & Dutch)
2.2. Table 2: The Netherlands Sub-regions
2.3. Figure 1: Age-gender distribution of the Dutch Disability Weight cohort sample *versus* the national population
2.4. Figure 2: Age-gender-education distribution of the Dutch Disability Weight cohort sample, by regions
2.5. Table 3: Test re-test analysis of the paired comparison, by NL-pooled and NL-region and educational level
2.6. Figure 4: Spearman’s correlation of probit coefficients by age, gender, highest attained level of education, chronic disease status, and region of residence
2.7. Table 4: Comparison of Dutch and European disability weights.

1. Statistical analysis: paired comparison

The paired comparison technique was used to derive disability weights for the Dutch population. In paired comparison technique, respondents are presented with two health state descriptions of hypothetical people (person A *versus* person B), each living in a particular health state, and then asked which person they regarded as being healthier over the other. A sample paired comparison question for two random health states was as follows:

****

Now we want to learn how people compare **different health problems**.

A person's health may limit how well parts of his body or his mind works. As a result, some people are not able to do all of the things in life that others may do, and some people are more severely limited than others.

Following is a <u>series</u> of questions about different health problems. In each question two different people will be described to you. You should imagine that these two people have the same number of years left to live, and that they will experience the health problems described for the rest of their lives. Identify which person you think **is healthier overall**, in terms of having fewer physical or mental limitations on what they can do in life.

Please note that there is no right or wrong answer to the following question. Instead, we are interested in finding out your personal views. Imagine that both people will have these problems for the rest of their lives.

Who do you think is <u>healthier overall</u>, the first person or the second person?

The first person has severe problems interacting with others and difficulty understanding simple questions or directions. The person has great difficulty with basic daily activities and becomes distressed by any change in routine.	The second person has headaches, dizziness, nausea and difficulty concentrating.

****

Paired comparison responses were analysed using a probit regression model. A binary response variable *Y* was coded as 1 (i.e. *Y* = 1) if the first health state in a pair was selected as the healthier one and -1 (i.e. *Y* = -1) if the second health state in a pair was selected as the healthier. Following the standard probit formulation, the model was given by: $P(Y = 1|X) = \Phi(X'\beta)$

where Φ is the cumulative distribution function of the standard normal distribution; *X* is a matrix of indicator variables; and parameters β represents probit regression coefficients which are estimated by maximum likelihood. For paired comparison responses involving *k* health states, we define *X* as a matrix of k -1 indicator variables for all except one health state; the indicator variable takes the value 1 if the state is chosen as the healthier option in a paired comparison, −1 if the state is the non-chosen alternative, and 0 for all states other than the pair being considered.

2. Supplemental tables and figures

2.1. Table 1: Brief lay descriptions for health states and their origins (in English (ENG) & Dutch (NL))

ID	Source	Health state	Lay description (ENG)	Lay description (NL)
		**Infectious disease**		
1	GBD 2010	Infectious disease: acute episode, mild	has a low fever and mild discomfort, but no difficulty with daily activities.	heeft lage koorts en licht ongemak, maar geen problemen met het uitvoeren van de dagelijkse activiteiten.
2	GBD 2010	Infectious disease: acute episode, moderate	has a fever and aches, and feels weak, which causes some difficulty with daily activities.	heeft koorts en pijn, voelt zich zwak, wat zorgt voor wat problemen bij het uitvoeren van dagelijkse activiteiten.
3	GBD 2010	Infectious disease: acute episode, severe	has a high fever and pain, and feels very weak, which causes great difficulty with daily activities.	heeft hoge koorts en pijn, voelt zich erg zwak, wat zorgt voor grote problemen bij het uitvoeren van de dagelijkse activiteiten.
4	*New*	Diarrhoea: with complications	has fever, nausea, abdominal cramps and diarrhea three or more times. Dehydration leaves this person weak and confused, causing great difficulty in daily activities.	heeft koorts, misselijkheid, buikkrampen en drie keer of vaker last van diarree. Door uitdroging is deze persoon slap en verward, wat veel moeite met dagelijkse activiteiten veroorzaakt.
5	GBD 2010	Diarrhoea: without complications	has diarrhea three or more times a day with occasional discomfort in the belly.	heeft drie keer of vaker per dag last van diarree en af en toe een vervelend gevoel in de buik.
6	GBD 2010	HIV/AIDS: receiving antiretroviral treatment	has occasional fevers and infections. The person takes daily medication that sometimes causes diarrhea.	heeft af en toe koorts en ontstekingen. De persoon neemt dagelijks medicatie die soms diarree veroorzaakt.
7	GBD 2010	AIDS cases: not receiving antiretroviral treatment	has severe weight loss, weakness, fatigue, cough and fever, and frequent infections, skin rashes and diarrhea.	heeft veel gewicht verloren en is zwak en moe. Deze persoon heeft ook last van hoesten en koorts en heeft regelmatig ontstekingen, huiduitslag en diarree.
8	GBD 2010	Tuberculosis: with HIV infection	has a persistent cough and fever, shortness of breath, night sweats, weakness and fatigue and severe weight loss.	heeft koorts en een aanhoudende hoest, is kortademig, voelt zich zwak en heeft veel gewicht verloren, wat veel moeite met dagelijkse activiteiten veroorzaakt.
9	*New*	Acute upper respiratory infections	has a stuffy nose, sore throat, and mild fever.	heeft een verstopte neus, keelpijn, en lichte koorts.
		**Cancer**		
10	GBD 2010	Cancer, diagnosis and primary therapy	has pain, nausea, fatigue, weight loss and high anxiety.	heeft pijn, is misselijk, moe, heeft gewicht verloren en is erg angstig.
11	GBD 2010	Cancer, metastatic	has severe pain, extreme fatigue, weight loss and high anxiety.	heeft ernstige pijn, is extreem moe, heeft gewicht verloren en is erg angstig en heeft moeite met het uitvoeren van dagelijkse activiteiten.
12	GBD 2010	Mastectomy	had one of her breasts removed and sometimes has pain or swelling in the arms.	heeft een borstamputatie ondergaan en heeft soms pijn en zwelling in de armen.

13	GBD 2010	Stoma	has a pouch attached to an opening in the belly to collect and empty stools.	heeft een zakje verbonden met een opening in de buik om uitwerpselen te verzamelen en af te voeren.
14	GBD 2010	Terminal phase, with medication (for cancers, end-stage kidney/liver disease)	has lost a lot of weight and regularly uses strong medication to avoid constant pain. The person has no appetite, feels nauseous, and needs to spend most of the day in bed.	heeft veel gewicht verloren en gebruikt regelmatig sterke medicatie om constante pijn te voorkomen. De persoon heeft geen eetlust, is misselijk en moet het grootste deel van de dag in bed doorbrengen.
15	GBD 2010	Terminal phase, without medication (for cancers, end-stage kidney/liver disease)	has lost a lot of weight and has constant pain. The person has no appetite, feels nauseous, and needs to spend most of the day in bed.	heeft veel gewicht verloren en heeft constant pijn. Deze persoon heeft geen eetlust, is misselijk en moet het grootste deel van de dag in bed doorbrengen.
16	Japanese (modified)	Cancer: residual stage, after treatment	decreased physical strength, feels tired and sometimes has abdominal pain or swelling of upper/lower limb and is sometimes anxious.	heeft verminderde spierkracht, is moe en heeft soms buikpijn of zwelling van de armen of benen en is soms angstig.
		**Cardiovascular and circulatory disease**		
17	GBD 2010	Acute myocardial infarction: days 1-2	has severe chest pain that becomes worse with any physical activity. The person feels nauseous, short of breath, and very anxious.	heeft ernstige pijn die erger wordt bij fysieke inspanning. De persoon voelt zich misselijk, kortademig en erg angstig.
18	GBD 2010	Cardiac conduction disorders and cardiac dysrhythmias	has periods of rapid and irregular heartbeats and occasional fainting.	heeft regelmatig last van een snelle en onregelmatige hartslag en valt soms flauw.
19	GBD 2010	Heart failure: mild	is short of breath and easily tires with moderate physical activity, such as walking uphill or more than a quarter-mile on level ground. The person feels comfortable at rest or during activities requiring less effort.	is kortademig en wordt snel moe tijdens het uitvoeren van matige fysieke activiteit, zoals bergop lopen of meer dan een halve kilometer lopen op vlakke grond. De persoon voelt zich op zijn gemak in rust of tijdens activiteiten die minder inspanning vergen.
20	GBD 2010	Heart failure: moderate	is short of breath and easily tires with minimal physical activity, such as walking only a short distance. The person feels comfortable at rest but avoids moderate activity.	is kortademig en wordt snel moe tijdens het uitvoeren van minimale fysieke activiteit, zoals een zeer korte afstand lopen. De persoon voelt zich op zijn gemak in rust, maar vermijdt activiteiten die inspanning vergen.
21	GBD 2010	Heart failure: severe	is short of breath and feels tired when at rest. The person avoids any physical activity, for fear of worsening the breathing problems.	is kortademig en is moe in rust. De persoon vermijdt elke fysieke activiteit, uit angst voor verergering van de ademhalingsproblemen.
22	GBD 2010	Stroke: long-term consequences, mild	has some difficulty in moving around and some weakness in one hand, but is able to walk without help.	heeft wat moeite met rondlopen en het bewegen van één hand, maar kan zonder hulp lopen.
23	GBD 2010	Stroke: long-term consequences, moderate	has some difficulty in moving around, and in using the hands for lifting and holding things, dressing and grooming.	heeft wat moeite met bewegen en rondlopen en kan de handen minder goed bewegen, waardoor deze persoon moeite heeft met het optillen en vasthouden van dingen, aankleden en verzorgen van zichzelf.

24	GBD 2010	Stroke: long-term consequences, severe	is confined to bed or a wheelchair, has difficulty speaking and depends on others for feeding, toileting and dressing.	is aan bed of een rolstoel gebonden, is afhankelijk van anderen voor eten, naar het toilet gaan en aankleden en heeft moeite met spreken.
		**Diabetes, digestive, and genitourinary disease**		
25	GBD 2010	Diabetic foot	has a sore on the foot that is swollen and causes some difficulty in walking.	heeft een ontstoken wond op de voet en de voet is opgezwollen. Dit veroorzaakt enig moeite met lopen.
26	GBD 2010	Diabetic neuropathy	has pain, tingling and numbness in the arms, legs, hands and feet. The person sometimes gets cramps and muscle weakness.	heeft pijn, tintelingen en doofheid in armen, benen, handen en voeten. De persoon heeft soms krampen en spiermoeheid.
27	*New*	Diabetes: without complications	is sometimes tired and has to take medication every day.	is soms moe en moet dagelijks medicijnen slikken.
28	*New*	Chronic kidney disease (stage III)	tires easily and sometimes has headaches.	wordt snel moe en heeft soms hoofdpijn.
29	GBD 2010	Chronic kidney disease (stage IV)	tires easily, has nausea, reduced appetite and difficulty sleeping.	wordt snel moe, is misselijk, heeft een verminderde eetlust en slaapproblemen.
30	GBD 2010	End-stage renal disease: on dialysis	is tired and has itching, cramps, headache, joint pains and shortness of breath. The person needs intensive medical care every other day lasting about half a day.	is moe en heeft last van jeuk, krampen, hoofdpijn, gewrichtspijn en kortademigheid. De persoon heeft om de dag intensieve medische zorg nodig die ongeveer een halve dag duurt.
31	GBD 2010	Decompensated liver cirrhosis	has a swollen belly and swollen legs. The person feels weakness, fatigue and loss of appetite.	heeft een gezwollen buik en gezwollen benen. De persoon voelt zwakte, vermoeidheid en verlies van eetlust en heeft problemen met het uitvoeren van dagelijkse activiteiten.
32	EURO	Urinary incontinence	cannot control urinating.	heeft geen controle over urine en is daardoor incontinent.
		**Respiratory diseases**		
33	GBD 2010	Asthma, controlled	has wheezing and cough once a month, which does not cause difficulty with daily activities.	moet eens per maand hoesten en is een beetje kortademig. Deze persoon heeft geen problemen met het uitvoeren van dagelijkse activiteiten.
34	GBD 2010	Asthma, partially controlled	has wheezing and cough once a week, which causes some difficulty with daily activities.	moet eens per week hoesten en is kortademig, waardoor deze persoon enkele problemen heeft met het uitvoeren van dagelijkse activiteiten.
35	GBD 2010	Asthma, uncontrolled	has wheezing, cough and shortness of breath more than twice a week, which causes difficulty with daily activities and sometimes wakes the person at night.	heeft meer dan twee keer per week een piepende ademhaling, hoesten en is dan kortademig, waardoor de persoon moeite heeft met dagelijkse activiteiten en soms 's nachts wakker wordt.
36	GBD 2010	COPD and other chronic respiratory disease, mild	has cough and shortness of breath after heavy physical activity, but is able to walk long distances and climb stairs.	moet hoesten en is kortademig na zware fysieke inspanning, maar deze persoon is wel in staat om lange afstanden te lopen en de trap op te lopen.

37	GBD 2010	COPD and other chronic respiratory disease, moderate	has cough, wheezing and shortness of breath, even after light physical activity. The person feels tired and can walk only short distances or climb only a few stairs.	moet hoesten en is kortademig, zelfs na lichte fysieke inspanning. De persoon is moe en kan alleen korte afstanden lopen of een paar traptreden oplopen.
38	GBD 2010	COPD and other chronic respiratory disease, severe	has cough, wheezing and shortness of breath all the time. The person has great difficulty walking even short distances or climbing any stairs, feels tired when at rest, and is anxious.	moet hoesten en is altijd kortademig. Deze persoon heeft veel moeite met het lopen van zeer korte afstanden of het oplopen van enkele traptreden, voelt zich ook tijdens het rusten moe en angstig.
		**Neurological disorders**		
39	GBD 2010	Dementia: mild	has some trouble remembering recent events, and finds it hard to concentrate and make decisions and plans.	heeft wat moeite met het onthouden van recente gebeurtenissen en vindt het moeilijk om zich te concentreren en beslissingen te nemen.
40	GBD 2010	Dementia: moderate	has memory problems and confusion, feels disoriented, at times hears voices that are not real, and needs help with some daily activities.	heeft geheugenproblemen en is verward, voelt zich gedesoriënteerd, hoort soms stemmen die niet echt zijn en heeft hulp nodig bij sommige dagelijkse activiteiten.
41	GBD 2010	Dementia: severe	has complete memory loss; no longer recognizes close family members; and requires help with all daily activities.	heeft volledig geheugenverlies, herkent naaste familieleden niet meer en heeft hulp nodig bij alle dagelijkse activiteiten en zelfzorg.
42	*New*	Headache: migraine, mild	has a throbbing headache and nausea causing difficulty with daily activities. Movement, light and noise worsen headaches and nausea.	heeft een kloppende hoofdpijn en misselijkheid wat moeite met dagelijkse activiteiten veroorzaakt. Bewegen, licht en geluid verergeren de hoofdpijn en misselijkheid.
43	GBD 2010	Headache: migraine, severe	has severe, throbbing head pain and nausea that cause great difficulty in daily activities and sometimes confine the person to bed. Moving around, light, and noise make it worse.	heeft ernstige, kloppende hoofdpijn en misselijkheid wat grote moeite met dagelijkse activiteiten veroorzaakt. Bewegen, licht en geluid verergeren de hoofdpijn en misselijkheid.
44	GBD 2010	Headache: tension-type	has a moderate headache that also affects the neck, which causes difficulty in daily activities.	heeft hoofdpijn en last van de nek. Dit veroorzaakt problemen bij het uitvoeren van dagelijkse activiteiten.
45	GBD 2010	Multiple sclerosis: mild	has mild loss of feeling in one hand, is a little unsteady while walking, has slight loss of vision in one eye, and often needs to urinate urgently.	heeft een verminderd gevoel in de handen, is wat onstabiel tijdens het lopen, kan wat minder goed zien en moet vaak dringend plassen.
46	GBD 2010	Multiple sclerosis: moderate	needs help walking, has difficulty with writing and arm coordination, has loss of vision in one eye and cannot control urinating.	heeft hulp nodig tijdens het lopen, heeft moeite met schrijven en het bewegen van de armen, kan minder goed zien en heeft geen controle over het plassen.
47	GBD 2010	Multiple sclerosis: severe	has slurred speech and difficulty swallowing. The person has weak arms and hands, very limited and stiff leg movement, has loss of vision in both eyes and cannot control urinating.	praat met dubbele tong en heeft moeite met slikken. Deze persoon heeft spierzwakte in armen en handen, erg beperkte en stijve beweging van de benen, verlies van zicht en heeft geen controle over plassen.
48	EURO	Idiopathic epilepsy: less severe (seizures <12 per year)	has sudden seizures two to five times a year, with violent muscle contractions and stiffness, loss of consciousness, and loss of urine or bowel control.	heeft twee tot vijf keer per jaar plotselinge toevallen, met ernstige spiersamentrekkingen en spierstijfheid, verlies van bewustzijn en verlies van controle over urine of darmen.

49	EURO	Idiopathic epilepsy: severe (seizures ≥ 1 per month)	has sudden seizures one or more times each month, with violent muscle contractions and stiffness, loss of consciousness, and loss of urine or bowel control. Between seizures the person has memory loss and difficulty concentrating.	heeft eens per maand of vaker last van plotselinge toevallen, met ernstige spiersamentrekkingen en spierstijfheid, verlies van bewustzijn en verlies van controle over urine of darmen. In de periode tussen de toevallen heeft deze persoon last van geheugenverlies en concentratieproblemen.
50	GBD 2010	Parkinson's disease: mild	has mild tremors and moves a little slowly, but is able to walk and do daily activities without assistance.	heeft last van milde tremors en beweegt wat langzaam, maar kan zonder hulp lopen en de dagelijkse activiteiten uitvoeren.
51	GBD 2010	Parkinson's disease: moderate	has moderate tremors and moves slowly, which causes some difficulty in walking and daily activities. The person has some trouble swallowing, talking, sleeping, and remembering things.	heeft matige tremors en beweegt langzaam, wat zorgt voor wat problemen tijdens het lopen en het uitvoeren van de dagelijkse activiteiten. De persoon heeft wat moeite met slikken, praten, slapen en het onthouden van dingen.
52	GBD 2010	Parkinson's disease: severe	has severe tremors and moves very slowly, which causes great difficulty in walking and daily activities. The person falls easily and has a lot of difficulty talking, swallowing, sleeping, and remembering things.	heeft ernstige tremors en beweegt erg langzaam, wat zorgt voor veel problemen tijdens het lopen en het uitvoeren van de dagelijkse activiteiten. De persoon valt regelmatig en heeft veel moeite met praten, slikken, slapen en het onthouden van dingen.
		**Mental, behavioural, and substance use disorders**		
53	GBD 2010	Alcohol use disorder: mild	drinks a lot of alcohol and sometimes has difficulty controlling the urge to drink. While intoxicated, the person has difficulty performing daily activities.	drinkt veel alcohol en heeft soms moeite om de drang om te drinken onder controle te houden. Als deze persoon dronken is, dan heeft hij of zij moeite met het uitvoeren van dagelijkse activiteiten.
54	GBD 2010	Alcohol use disorder: severe	gets drunk almost every day and is unable to control the urge to drink. Drinking and recovering replace most daily activities. The person has difficulty thinking, remembering and communicating, and feels constant pain and fatigue.	wordt bijna elke dag dronken en is niet in staat om de drang om te drinken te beheersen. Drinken en herstellen van het drinken vervangen de meeste dagelijkse activiteiten. De persoon heeft moeite met denken, onthouden en communiceren en heeft constant pijn en is constant moe.
55	*New*	Drug dependence: mild	uses drugs at least once a week and has difficulty controlling this habit. When not using drugs, then this person can function normally.	gebruikt ten minste een keer per week drugs en heeft moeite deze gewoonte onder controle te houden. Als deze persoon geen drugs gebruikt, dan kan deze persoon normaal functioneren.
56	*New*	Drug dependence: moderate to severe	uses drugs at least once daily and has difficulty controlling addiction. The person sometimes has depression, hallucinations and mood swings and has difficulty with daily activities.	gebruikt dagelijks drugs en heeft moeite om de verslaving onder controle te houden. De persoon heeft soms depressies, hallucinaties en stemmingswisselingen en heeft moeite met dagelijkse activiteiten.

57	GBD 2010	Anxiety disorders: mild	feels mildly anxious and worried, which makes it slightly difficult to concentrate, remember things, and sleep. The person tires easily but is able to perform daily activities.	voelt zich een beetje gespannen en angstig, wat het moeilijker maakt om zich te concentreren, dingen te onthouden en te slapen. De persoon raakt snel vermoeid, maar kan de dagelijkse activiteiten uitvoeren.
58	GBD 2010	Anxiety disorders: moderate	feels anxious and worried, which makes it difficult to concentrate, remember things, and sleep. The person tires easily and finds it difficult to perform daily activities.	voelt zich gespannen en angstig, wat het moeilijk maakt om zich te concentreren, dingen te onthouden en te slapen. Deze persoon is snel moe en heeft moeite met het uitvoeren van de dagelijkse activiteiten.
59	GBD 2010	Anxiety disorders: severe	constantly feels very anxious and worried, which makes it difficult to concentrate, remember things and sleep. The person has lost pleasure in life and thinks about suicide.	voelt zich doorlopend erg gespannen en angstig, wat het moeilijk maakt zich te concentreren, dingen te onthouden en te slapen. De persoon heeft het plezier in het leven verloren en denkt over zelfmoord.
60	GBD 2010	Major depressive disorder: mild episode	feels persistent sadness and has lost interest in usual activities. The person can still function in daily life with extra effort, but sleeps badly, feels tired, and has trouble concentrating.	voelt zich aanhoudend somber en heeft geen interesse meer in dagelijkse bezigheden. Deze persoon slaapt soms slecht, voelt zich moe of heeft moeite zich te concentreren, maar slaagt er met extra inspanning toch in om in het dagelijks leven te functioneren.
61	GBD 2010	Major depressive disorder: moderate episode	has constant sadness and has lost interest in usual activities. The person has some difficulty in daily life, sleeps badly, has trouble concentrating, and sometimes thinks about harming himself (or herself).	voelt zich doorlopend somber en neerslachtig en heeft geen interesse meer in dagelijkse bezigheden. Deze persoon heeft moeite met het dagelijkse leven, slaapt slecht, heeft moeite zich te concentreren en denkt er soms aan om zichzelf pijn te doen.
62	GBD 2010	Major depressive disorder: severe episode	has overwhelming, constant sadness and cannot function in daily life. The person sometimes loses touch with reality and wants to harm or kill himself (or herself).	voelt doorlopend een overweldigend gevoel van somberheid en neerslachtigheid, kan niet functioneren in het dagelijks leven. De persoon verliest vaak het contact met de werkelijkheid en wil zichzelf pijn doen of doden.
63	*New*	Burnout: minor complications	is tired, irritable, forgetful, and sometimes has trouble sleeping, difficulty thinking clearly and concentrating, and some difficulty performing daily activities.	is moe, prikkelbaar, vergeetachtig en heeft soms last van slaapproblemen, moeite met helder denken en concentreren en een beetje moeite om dagelijkse activiteiten uit te voeren.
64	*New*	Burnout: major complications	is extremely tired, very irritable, forgetful and has trouble sleeping, great difficulty thinking and concentrating clearly, and great difficulty performing daily activities.	is extreem moe, erg prikkelbaar, vergeetachtig en heeft last van slaapproblemen, veel moeite met helder denken en concentreren en veel moeite om dagelijkse activiteiten uit te voeren.
65	GBD 2010	Bipolar disorder: manic episode	is hyperactive, hears and believes things that are not real, and engages in impulsive and aggressive behavior that endanger the person and others.	is hyperactief, hoort en gelooft dingen die niet echt zijn en vertoont impulsief en agressief gedrag dat de persoon en anderen in gevaar brengt.

66	GBD 2010	Bipolar disorder: residual state	has mild mood swings, irritability and some difficulty with daily activities.	heeft soms stemmingswisselingen, is prikkelbaar en heeft enige moeite met het uitvoeren van dagelijkse activiteiten.
67	GBD 2010	Schizophrenia: acute state	hears and sees things that are not real and is afraid, confused, and sometimes violent. The person has great difficulty with communication and daily activities, and sometimes wants to harm or kill himself (or herself).	hoort en ziet dingen die niet echt zijn, is bang en verward en heeft veel moeite met communiceren. Deze persoon is erg vergeetachtig, heeft veel moeite met dagelijkse bezigheden en wil zichzelf pijn doen.
68	GBD 2010	Schizophrenia: residual state	hears and sees things that are not real and has trouble communicating. The person can be forgetful, has difficulty with daily activities, and thinks about hurting himself (or herself).	hoort en ziet dingen die niet echt zijn en heeft enige moeite met communiceren. De persoon kan vergeetachtig zijn, heeft enige moeite met dagelijkse activiteiten en denkt erover om zichzelf pijn te doen.
69	*New*	Anorexia nervosa	is very underweight and physically weak from starvation. Also has an overwhelming fear of weight gain and severe anxiety. May engage in uncontrolled overeating, vomiting, or excessive exercise.	heeft ernstig ondergewicht en is fysiek zwak door uithongering. Deze persoon heeft een grote angst voor gewichtstoename, is erg angstig, heeft soms eetbuien, braken, of beweegt overmatig.
70	*New*	Bulimia nervosa	experiences overwhelming concerns about body weight, shape, or eating, with frequent uncontrolled overeating, guilt and distress. Tries to compensate by fasting, vomiting, or excessive exercise.	ervaart grote zorgen over het lichaamsgewicht, lichaamsvorm of eten. Deze persoon heeft vaak ongecontroleerde eetbuien, schuldgevoelens, ervaart angst en probeert te compenseren door niks of zeer weinig te eten, braken of overmatige lichaamsbeweging.
71	*New*	Binge eating disorder	has frequent uncontrolled overeating with guilt, disgust, and distress, which can result in weight gain and interferes with daily life and relationships.	heeft vaak ongecontroleerde eetbuien en ervaart schuldgevoelens, walging en angst. De eetbuien kunnen gewichtstoename veroorzaken en problemen in het dagelijkse leven en sociale relaties.
72	*New*	Other specified feeding and eating disorder	engages in fasting, vomiting, excessive exercise, or uncontrolled overeating. Experiences overwhelming concerns about body weight, shape, or eating, and may experience severe anxiety.	eet niet of zeer weinig, braakt, beweegt overmatig of heeft ongecontroleerde eetbuien. Deze persoon ervaart grote zorgen over lichaamsgewicht, lichaamsvorm of eten, en kan erg angstig zijn.
73	*New*	Attention deficit hyperactivity disorder: mild	is hyperactive and has some difficulty concentrating, remembering things, and completing tasks. As a result, this person experiences some limitations in performing daily activities, at school and at work.	is hyperactief, heeft een beetje moeite met concentreren, onthouden en het afmaken van taken. Hierdoor ondervindt deze persoon enkele beperkingen tijdens het uitvoeren van dagelijkse activiteiten, op school en op het werk.
74	*New*	Attention deficit hyperactivity disorder: moderate	is hyperactive, has difficulty concentrating, remembering and completing tasks. As a result, this person experiences moderate limitations in performing daily activities, at school and at work.	is hyperactief, heeft moeite met concentreren, onthouden en het afmaken van taken. Hierdoor ondervindt deze persoon matige beperkingen tijdens het uitvoeren van dagelijkse activiteiten, op school en op het werk.

75	*New*	Attention deficit hyperactivity disorder: severe	is hyperactive, has great difficulty concentrating, remembering and completing tasks. As a result, this person experiences severe limitations in performing daily activities, at school and at work.	is hyperactief, heeft veel moeite met concentreren, onthouden en het afmaken van taken. Hierdoor ondervindt deze persoon ernstige beperkingen tijdens het uitvoeren van dagelijkse activiteiten, op school en op het werk.
76	*New*	Autism: moderate	has difficulty interacting with other people, and is slow to understand or respond to questions. The person is often preoccupied with one thing and has some difficulty with basic daily activities.	heeft moeite met interactie met andere mensen en is traag in het begrijpen of beantwoorden van vragen. Deze persoon is vaak met één ding bezig en heeft enige moeite met dagelijkse activiteiten.
77	GBD 2010	Autism: severe	has severe problems interacting with others and difficulty understanding simple questions or directions. The person has great difficulty with basic daily activities, is sensitive to loud noises and becomes distressed by any change in routine.	heeft ernstige problemen in de omgang met anderen en moeite met het begrijpen van eenvoudige vragen of aanwijzingen. De persoon heeft grote moeite met dagelijkse activiteiten, is gevoelig voor geluiden en raakt van streek door elke verandering in de routine.
78	GBD 2013	Intellectual disability: borderline (IQ 70/75-85/90)	is slow in learning at school. As an adult, the person has some difficulty doing complex or unfamiliar tasks but otherwise functions independently.	heeft moeite met leren op school. Als volwassene heeft deze persoon moeite met concentreren en het uitvoeren van moeilijke of onbekende taken.
79	GBD 2010	Intellectual disability: mild (IQ 50/55-70)	has low intelligence and is slow in learning at school. As an adult, the person can work at simple supervised jobs and live independently, but often needs help to raise children.	heeft een lage intelligentie en heeft veel moeite met leren op school. Als volwassene kan deze persoon eenvoudige taken onder toezicht uitvoeren en onafhankelijk wonen, maar deze persoon kan alleen onder begeleiding werken in eenvoudige banen en hij/zij heeft wel vaak hulp nodig met het opvoeden van kinderen.
80	GBD 2013	Intellectual disability: moderate (IQ 35/40-50/55)	has low intelligence and is slow in learning to speak and do simple tasks. As an adult, the person requires a lot of support to work productively, live independently and raise children.	heeft een lage intelligentie en heeft veel moeite met leren spreken en het aanleren van simpele taken. Als volwassene heeft deze persoon veel begeleiding en steun nodig om onafhankelijk te wonen. Deze persoon kan alleen onder begeleiding werken in de meest eenvoudige banen en heeft veel hulp en ondersteuning nodig bij het opvoeden van kinderen.
81	GBD 2013 (modified)	Intellectual disability: severe (IQ 20/25-35/40)	has very low intelligence and cannot speak more than a few sentences, needs constant supervision and help with most daily activities, and can do only the simplest tasks.	heeft een zeer lage intelligentie en kan alleen enkele simpele zinnen spreken. Deze persoon heeft constant toezicht en hulp nodig met de meest basale dagelijkse activiteiten en kan alleen onder toezicht zeer eenvoudige taken uitvoeren.
82	GBD 2013	Intellectual disability: profound (IQ less than 20-25)	has very low intelligence, has almost no language, and does not understand even the most basic requests or instructions. The person requires constant supervision and help for all activities.	heeft een zeer lage intelligentie en kan niet meer dan een paar woorden spreken en begrijpt zelfs de meest simpele instructies niet. Deze persoon heeft constant toezicht en hulp nodig bij alle dagelijkse activiteiten.
83	*New*	Personality disorders: mild	has unrealistically high or low self-worth. Experiences difficulties managing conflict in relationships, setting goals,	heeft een onrealistisch hoge of lage eigenwaarde. Deze persoon heeft moeite met conflicten in relaties, het

			and managing stress. Symptoms cause distress and difficulty in school or work.	stellen van doelen en het omgaan met stress. Hierdoor heeft deze persoon last van angst en moeilijkheden op school of op het werk.
84	*New*	Personality disorders: moderate	sees themselves as markedly superior/inferior. Frequently experiences or avoids conflict, with significant problems in relationships, goal setting, education, and employment. Experiences severe distress and occasionally harms themselves or others.	ziet zichzelf als duidelijk beter of slechter dan anderen en ervaart of vermijdt vaak conflicten, waardoor deze persoon veel problemen heeft met sociale relaties. Deze persoon heeft moeite met het stellen van doelen en met opleiding en werk, ervaart hevige angst en verwondt zichzelf soms.
85	*New*	Personality disorders: severe	experiences extreme self-hatred or inflated self-esteem. Has no purpose in life and cannot engage effectively with others. Experiences extreme distress in life, reckless behaviour, frequent violence, and severe self-harm.	heeft extreme zelfhaat of juist een opgeblazen gevoel van eigenwaarde. Deze persoon heeft geen doel in het leven en kan niet goed omgaan met anderen. Deze persoon ervaart zeer grote moeilijkheden in het leven en vertoont roekeloos gedrag, veelvuldig geweld en verwondt zichzelf.
		**Hearing and vision loss**		
86	EURO	Hearing loss: mild	has great difficulty hearing and understanding another person talking in a noisy place (for example, on an urban street).	heeft veel moeite met het verstaan van iemand anders die praat in een rumoerige omgeving (bijvoorbeeld op straat in de stad).
87	EURO	Hearing loss: moderate	is unable to hear and understand another person talking in a noisy place (for example, on an urban street), and has difficulty hearing another person talking even in a quiet place or on the phone.	kan iemand anders die praat in een luidruchtige omgeving (bijvoorbeeld op straat in de stad) niet verstaan en heeft moeite met het verstaan van iemand anders die praat als dit op een rustige plek is of aan de telefoon.
88	EURO (modified)	Hearing loss: severe	is unable to hear and understand another person talking, even in a quiet place, and unable to take part in a phone conversation. Difficulties with communicating and relating to others cause emotional impact at times (for example worry, depression or loneliness).	kan iemand anders niet verstaan, zelfs niet op een rustige plek en kan geen telefoongesprek voeren. Deze persoon heeft moeite met communiceren en sociale relaties en heeft daardoor vaak zorgen, depressie of gevoelens van eenzaamheid.
89	EURO	Hearing loss: profound	is unable to hear and understand another person talking, even in a quiet place, is unable to take part in a phone conversation, and has great difficulty hearing anything in any other situation. Difficulties with communicating and relating to others often cause worry, depression or loneliness.	kan iemand anders niet verstaan, zelfs niet op een rustige plek en kan geen telefoongesprek voeren. Deze persoon heeft veel moeite met horen in elke situatie. Deze persoon heeft moeite met communiceren en sociale relaties en heeft daardoor vaak zorgen, depressie of gevoelens van eenzaamheid.
90	EURO	Hearing loss: complete	cannot hear at all in any situation, including even the loudest sounds, and cannot communicate verbally or use a phone. Difficulties with communicating and relating to others often cause worry, depression or loneliness.	is volledig doof en kan in geen enkele situatie horen, zelfs niet de hardste geluiden. Deze persoon kan niet verbaal communiceren, zoals praten of bellen, en heeft grote moeite met sociale relaties en daardoor vaak zorgen, depressie of gevoelens van eenzaamheid.

91	EURO	Hearing loss: mild with ringing	has great difficulty hearing and understanding another person talking in a noisy place (for example, on an urban street), and sometimes has annoying ringing in the ears.	heeft veel moeite met het verstaan van iemand anders in een rumoerige omgeving (bijvoorbeeld op straat in de stad) en heeft soms last van een vervelende piep in de oren.
92	EURO	Hearing loss: moderate with ringing	is unable to hear and understand another person talking in a noisy place (for example, on an urban street), has difficulty hearing another person talking even in a quiet place or on the phone, and has annoying ringing in the ears for 5 minutes at a time, almost every day.	kan iemand anders die praat in een luidruchtige omgeving (bijvoorbeeld op straat in de stad) niet verstaan en heeft moeite met het verstaan van iemand anders die praat als dit op een rustige plek is of aan de telefoon, en heeft bijna elke dag, 5 minuten achter elkaar, last van een vervelende piep in de oren.
93	EURO (modified)	Hearing loss: severe with ringing	is unable to hear and understand another person talking, even in a quiet place, is unable to take part in a phone conversation, and has annoying ringing in the ears for more than 5 minutes at a time, almost every day. Difficulties with communicating and relating to others cause emotional impact at times (for example worry, depression or loneliness).	kan iemand anders niet verstaan, zelfs niet op een rustige plek en kan geen telefoongesprek voeren. Deze persoon heeft bijna elke dag, 5 minuten achter elkaar, last van een vervelende piep in de oren. Deze persoon heeft moeite met communiceren en met sociale relaties en heeft daardoor vaak zorgen, depressie of gevoelens van eenzaamheid.
94	EURO	Hearing loss: profound with ringing	is unable to hear and understand another person talking, even in a quiet place, is unable to take part in a phone conversation, has great difficulty hearing anything in any other situation, and has annoying ringing in the ears for more than 5 minutes at a time, several times a day. Difficulties with communicating and relating to others often cause worry, depression, or loneliness.	kan iemand anders niet verstaan, zelfs niet op een rustige plek, kan geen telefoongesprek voeren. Deze persoon heeft extreme moeite met het horen in elke situatie en heeft meerdere keren per dag, 5 minuten achter elkaar, last van een vervelende piep in de oren. Deze persoon heeft moeite met communiceren en sociale relaties en heeft daardoor vaak zorgen, depressie of gevoelens van eenzaamheid.
95	EURO	Hearing loss: complete with ringing	cannot hear at all in any situation, including even the loudest sounds, and cannot communicate verbally or use a phone, and has very annoying ringing in the ears for more than half of the day. Difficulties with communicating and relating to others often cause worry, depression or loneliness.	is volledig doof en kan in geen enkele situatie horen, zelfs niet de hardste geluiden, en heeft meer dan de helft van de dag een vervelende piep in de oren. Deze persoon kan niet verbaal communiceren, zoals praten of bellen, en heeft grote moeite met sociale relaties en daardoor vaak zorgen, depressie of gevoelens van eenzaamheid.
96	GBD 2010	Unilateral hearing loss	can hear well with one ear but has hearing loss in the other ear, resulting in some trouble following a conversation in a noisy environment.	kan goed horen met één oor, maar heeft gehoorverlies in het andere oor. Dit zorgt voor wat problemen tijdens het volgen van een gesprek in een luidruchtige omgeving.
97	GBD 2010	Distance vision: mild impairment	has some difficulty with distance vision, for example reading signs, but no other problems with eyesight.	heeft wat problemen met ver zien, bijvoorbeeld het lezen van verkeersborden, maar geen andere problemen met zien.
98	GBD 2010	Distance vision: moderate impairment	has vision problems that make it difficult to recognize faces or objects across a room.	heeft problemen met zien, waardoor deze persoon moeite heeft met het herkennen van gezichten of dingen aan de andere kant van een ruimte.

99	GBD 2010	Distance vision: severe impairment	has severe vision loss, which causes difficulty in daily activities, some emotional impact (for example worry), and some difficulty going outside the home without assistance.	heeft ernstige problemen met zien en dit veroorzaakt problemen met het uitvoeren van dagelijkse activiteiten. Deze persoon heeft enige zorgen en angsten en heeft wat moeite om buitenshuis te komen zonder hulp.
100	GBD 2010	Distance vision: blindness	is completely blind, which causes great difficulty in some daily activities, worry and anxiety, and great difficulty going outside the home without assistance.	is volledig blind, waardoor deze persoon veel problemen heeft bij uitvoeren van dagelijkse activiteiten. Deze persoon heeft zorgen en angsten en veel problemen om buitenshuis komen zonder hulp.
101	GBD 2013	Distance vision: monocular impairment	is blind in one eye and has difficulty judging distances.	is blind aan één oog en heeft moeite met het inschatten van afstanden.
102	GBD 2010	Near vision impairment	has difficulty seeing things that are nearer than 3 feet, but has no difficulty with seeing things at a distance.	heeft moeite met het zien van dingen op een korte afstand tot een meter, maar heeft geen moeite met het zien van dingen die ver weg zijn.
		**Musculoskeletal disorders**		
103	GBD 2013	Low back pain: mild	has mild back pain, which causes some difficulty dressing, standing, and lifting things.	heeft lichte rugpijn, waardoor deze persoon moeite heeft met aankleden, staan en optillen van dingen.
104	GBD 2013	Low back pain: moderate	has moderate back pain, which causes difficulty dressing, sitting, standing, walking, and lifting things.	heeft matige rugpijn, waardoor het moeilijk is om zich aan te kleden, te zitten, te staan, te lopen en dingen op te tillen.
105	GBD 2013	Low back pain: severe (without leg pain)	has severe back pain, which causes difficulty dressing, sitting, standing, walking, and lifting things. The person sleeps poorly and feels worried.	heeft ernstige rugpijn, waardoor het moeilijk is om zich aan te kleden, te zitten, te staan, te lopen en dingen op te tillen. De persoon slaapt slecht en voelt zich bezorgd.
106	GBD 2013	Neck pain: mild	has neck pain, and has difficulty turning the head and lifting things.	heeft nekpijn en heeft moeite het hoofd te draaien en dingen op te tillen.
107	GBD 2013	Neck pain: moderate	has constant neck pain, and has difficulty turning the head, holding arms up, and lifting things	heeft doorlopend nekpijn en heeft moeite het hoofd te draaien, armen op te tillen en dingen op te tillen.
108	GBD 2013	Neck pain: severe	has severe neck pain, and difficulty turning the head and lifting things. The person gets headaches and arm pain, sleeps poorly, and feels tired and worried.	heeft hevige nekpijn en moeite met het draaien van het hoofd en het optillen van dingen. Deze persoon heeft ook hoofdpijn en pijn in de armen, slaapt slecht en voelt zich moe en bezorgd.
109	GBD 2010	Musculoskeletal problems, lower limbs: moderate	has moderate pain in the leg, which makes the person limp, and causes some difficulty walking, standing, lifting and carrying heavy things, getting up and down and sleeping.	heeft matige pijn in de benen, wat ervoor zorgt dat de persoon mank loopt en problemen heeft met lopen, staan, tillen en het dragen van zware dingen, omhoog en naar beneden komen en slapen.
110	GBD 2010	Musculoskeletal problems, lower limbs: severe	has severe pain in the leg, which makes the person limp and causes a lot of difficulty walking, standing, lifting and carrying heavy things, getting up and down, and sleeping.	heeft ernstige pijn in de benen, wat ervoor zorgt dat de persoon mank is en problemen heeft met lopen, staan, tillen en het dragen van zware dingen, omhoog en naar beneden komen en slapen.

111	GBD 2010	Musculoskeletal problems, upper limbs: moderate	has moderate pain and stiffness in the arms and hands, which causes difficulty lifting, carrying, and holding things, and trouble sleeping because of the pain.	heeft een matige pijn en stijfheid in de armen en handen, wat zorgt voor problemen met het tillen, dragen en vasthouden van dingen en problemen met slapen vanwege de pijn.
112	GBD 2010	Musculoskeletal problems: generalized, moderate	has pain and deformity in most joints, causing difficulty moving around, getting up and down, and using the hands for lifting and carrying. The person often feels fatigue.	heeft pijn en misvormingen in de meeste gewrichten, waardoor deze persoon moeite heeft met bewegen en rondlopen, het uitvoeren van dagelijkse activiteiten en slapen. Deze persoon is vaak moe.
113	GBD 2010	Musculoskeletal problems: generalized, severe	has severe, constant pain and deformity in most joints, causing difficulty moving around, getting up and down, eating, dressing, lifting, carrying and using the hands. The person often feels sadness, anxiety and extreme fatigue.	heeft ernstige, aanhoudende pijn en misvormingen in de meeste gewrichten, waardoor het moeilijk is om te bewegen, rond te lopen, te eten, zichzelf aan te kleden, dingen op te tillen en de handen te gebruiken. Deze persoon voelt zich vaak verdrietig en angstig en heeft last van extreme vermoeidheid.
114	*New*	Gout: acute, mild	has some swelling and pain in the leg, causing this person to have a little difficulty walking.	heeft wat zwelling en pijn in een gewricht, waardoor deze persoon een beetje moeite heeft om te lopen.
115	*New*	Gout: acute, moderate	has moderate swelling and pain in the leg, causing this person to have difficulty getting up and walking around.	heeft matige zwelling en pijn in een gewricht, waardoor deze persoon moeite heeft met opstaan en rondlopen.
116	GBD 2013	Gout: acute, severe	has severe pain and swelling in the leg, making it very difficult to get up and down, stand, walk, lift, and carry heavy things. The person has trouble sleeping because of the pain.	heeft hevige pijn en zwelling in een gewricht, waardoor het erg moeilijk is om te staan, te lopen en dingen op te tillen. De persoon heeft moeite met slapen vanwege de pijn.
		**Injury**		
117	GBD 2010	Amputation of finger(s), excluding thumb	has lost a finger of one hand. At times there is pain and tingling in the stump.	mist door amputatie een vinger van een hand. Soms heeft deze persoon pijn en een tintelend gevoel in de stomp.
118	GBD 2010	Amputation of thumb: long term	has lost one thumb, causing some difficulty in using the hand, pain, and tingling in the stump.	heeft door amputatie geen duim meer. Dit leidt tot enige moeite met het gebruiken van de hand, pijn en een tinteling in de stomp.
119	GBD 2013	Amputation of one upper limb: long term, with treatment	has lost one hand and part of the arm, leaving pain and tingling in the stump. The person has an artificial arm that makes it possible to lift objects and do daily activities such as cooking, with some extra effort.	mist door amputatie een hand en deel van de arm. Dit leidt tot pijn en een tintelend gevoel in de stomp. De persoon heeft een kunstarm die het mogelijk maakt om objecten op te tillen en met wat extra inspanning dagelijkse activiteiten uit te voeren, zoals koken.
120	GBD 2013	Amputation of one upper limb: long term, without treatment	has lost one hand and part of the arm, leaving pain and tingling in the stump. The person needs help from others to lift objects or do daily activities such as cooking.	mist door amputatie een hand en deel van de arm. Dit leidt tot pijn en een tintelend gevoel in de stomp. De persoon heeft hulp nodig van anderen om dagelijkse activiteiten uit te voeren, zoals koken.
121	GBD 2013	Amputation of both upper limbs: long term, with treatment	has lost part of both arms, leaving pain and tingling in the stumps. The person has two artificial arms that make it	mist door amputatie een deel van beide armen. Dit leidt tot pijn en een tintelend gevoel in de stompen. De

			possible to do daily activities, with a great deal of extra effort.	persoon heeft twee kunstarmen die het mogelijk maken om dagelijkse activiteiten uit te voeren, met veel extra inspanning.
122	GBD 2013	Amputation of both upper limbs: long term, without treatment	has lost part of both arms, leaving pain and tingling in the stumps. The person needs a great deal of help from others to do even basic daily activities such as eating and using the toilet, and the person is very limited in other activities.	mist door amputatie een deel van beide armen. Dit zorgt voor pijn en een tintelend gevoel in de stompen. De persoon heeft veel hulp nodig van anderen, zelfs bij dagelijkse activiteiten, zoals eten en naar het toilet gaan, en de persoon is erg beperkt in andere activiteiten.
123	GBD 2010	Amputation of toe	has lost one toe, leaving occasional pain and tingling in the stump.	mist door amputatie een teen. De persoon heeft nu af en toe pijn en een tintelend gevoel in de stomp.
124	GBD 2013	Amputation of one lower limb: long term, with treatment	has lost part of one leg, leaving pain and tingling in the stump. The person has an artificial leg that helps in moving around.	mist door amputatie een deel van een been. Dit leidt tot pijn en een tinteling in de stomp. De persoon heeft een kunstbeen, waardoor deze persoon wel kan lopen.
125	GBD 2013	Amputation of one lower limb: long term, without treatment	has lost part of one leg, leaving pain and tingling in the stump. The person does not have an artificial leg, has frequent sores, and uses crutches.	mist door amputatie een deel van een been. Dit leidt tot pijn en een tinteling in de stomp. De persoon heeft geen kunstbeen, heeft regelmatig ontstoken wonden aan de stomp en gebruikt krukken.
126	GBD 2013	Amputation of both lower limbs: long term, with treatment	has lost part of both legs, leaving pain and tingling in the stumps. The person has two artificial legs that make moving around possible, with extra effort.	mist door amputatie een deel van beide benen. Dit leidt tot pijn en een tintelend gevoel in de stompen. De persoon heeft twee kunstbenen die het mogelijk maken om met extra inspanning te lopen.
127	GBD 2013	Amputation of both lower limbs: long term, without treatment	has lost part of both legs, leaving pain, tingling, and frequent sores in the stumps. The person has great difficulty moving around, has episodes of depression and anxiety, and needs help from others to do many daily activities.	mist door amputatie een deel van beide benen. Dit leidt tot pijn, een tintelend gevoel en regelmatig ontstoken wonden in de stompen. De persoon heeft veel moeite met bewegen en heeft af en toe last van depressie en angsten en heeft bij veel dagelijkse activiteiten hulp van anderen nodig.
128	GBD 2010	Burns, <20% total burned surface area without lower airway burns: short term, with or without treatment	has a burn on part of the body. Parts of the burned area are painful, and other parts have lost feeling.	heeft een pijnlijke brandwond op een deel van het lichaam. In delen van de verbrande huid zit geen gevoel meer.
129	GBD 2010	Burns, <20% total burned surface area or <10% total burned surface area if head/neck or hands/wrist involved: long term, with or without treatment	has scars caused by a burn. The scars are sometimes painful and itchy.	heeft littekens die veroorzaakt zijn door een brandwond. De littekens zijn soms pijnlijk en jeuken.
130	GBD 2010	Burns, ≥20% total burned surface area: short term, with or without treatment	has a painful burn over a large part of the body. Parts of the burned area have lost feeling, and the person feels anxious and unwell.	heeft een pijnlijke brandwond op een groot deel van het lichaam. In delen van de verbrande huid zit geen gevoel meer. De persoon voelt zich nerveus en onwel.

131	GBD 2010	Burns, ≥20% total burned surface area or ≥10% total burned surface area if head/neck or hands/wrist involved: long term, with treatment	has scars caused by burns over a large part of the body. The scars are frequently painful and itchy, and the person is often sad.	heeft littekens van brandwonden op een groot deel van het lichaam. De littekens voelen vaak pijnlijk aan en jeuken en de persoon is vaak verdrietig.
132	GBD 2010	Burns, ≥20% total burned surface area or ≥10% total burned surface area if head/neck or hands/wrist involved: long term, without treatment	has severe, disfiguring and itchy scars caused by burns over a large part of the body. The person cannot move some joints, feels sad, and has great difficulty with self-care such as dressing and toileting.	heeft ernstige, misvormende en jeukende littekens veroorzaakt door brandwonden op een groot deel van het lichaam. De persoon kan bepaalde gewrichten niet bewegen, voelt zich verdrietig en heeft veel moeite om voor zichzelf te zorgen, bijvoorbeeld tijdens het aankleden en naar het toilet gaan.
133	GBD 2010	Crush injury: short or long term, with or without treatment	had part of the body crushed, leaving pain, swelling, tingling and limited feeling in the affected area	heeft een arm of een been verbrijzeld. Dit leidt tot pijn, zwellingen, een tintelend gevoel en een verminderd gevoel in dit lichaamsdeel.
134	GBD 2013	Dislocation of hip: long term, with or without treatment	walks with a limp and feels discomfort when walking.	loopt mank en voelt pijn tijdens het lopen.
135	GBD 2013	Dislocation of knee: long term, with or without treatment	has a knee out of joint, causing pain and difficulty moving the knee, which sometimes gives way. The person needs crutches for walking and help with self-care such as dressing.	heeft een knie die uit de kom geschoten is. Dit leidt tot veel pijn en moeite met het bewegen van de knie. De persoon heeft krukken nodig om te lopen en hulp nodig bij zelfzorg, bijvoorbeeld met aankleden.
136	GBD 2010	Dislocation of shoulder: long term, with or without treatment	has a shoulder that is out of joint, causing pain and difficulty moving. The person has difficulty with daily activities such as dressing and cooking.	heeft de schouder uit de kom geschoten. Dit leidt tot veel pijn en moeite met bewegen. De persoon heeft moeite met dagelijkse activiteiten, zoals aankleden en koken.
137	*New*	Other injuries of muscle and tendon (includes sprains, strains and dislocations other than shoulder, knee, hip)	has an overworked muscle that causes pain and swelling.	heeft een overbelaste spier die zorgt voor pijn en zwellingen.
138	GBD 2010	Drowning and nonfatal submersion: short or long term, with or without treatment	has breathlessness, anxiety, cough, and vomiting.	raakt buiten adem, moet hoesten en overgeven en is angstig.
139	GBD 2010	Fracture of clavicle, scapula or humerus: short or long term, with or without treatment	has a broken shoulder bone, which is painful and swollen. The person cannot use the affected arm and has difficulty with getting dressed.	heeft een gebroken schouder, die pijnlijk en opgezwollen is. De persoon kan de arm niet gebruiken en heeft moeite met het aankleden.
140	GBD 2010	Fracture of foot bones: short term, with or without treatment	has a broken foot bone, which causes pain, swelling, and difficulty walking.	heeft een gebroken voet. Deze persoon heeft pijnlijke en gezwollen voet en heeft moeite met lopen.
141	GBD 2013	Fracture of foot bones: long term, without treatment	had a broken foot in the past that did not heal properly. The person now has pain in the foot and has some difficulty walking.	heeft in het verleden een gebroken voet gehad die niet volledig geheeld is. De persoon heeft nu pijn in de voet en moeite met lopen.

142	*New*	Fracture of hand: short term, with or without treatment	has a broken hand. This person has a painful and swollen hand and has some difficulty with self-care, such as getting dressed.	heeft een gebroken hand. Deze persoon heeft een pijnlijke en gezwollen hand en heeft een beetje moeite met zelfzorg, zoals aankleden.
143	GBD 2010	Fracture of hand: long term, without treatment	has stiffness in the hand and a weak grip.	heeft last van stijfheid en krachtverlies van de hand.
144	GBD 2010	Fracture of neck of femur: short term, with or without treatment	has broken a hip and is in pain. The person cannot stand or walk, and needs help washing, dressing, and going to the toilet.	heeft een gebroken heup en heeft veel pijn. De persoon kan niet staan of lopen, heeft hulp nodig met het wassen, aankleden en naar het toilet gaan.
145	GBD 2010	Fracture of neck of femur: long term, with treatment	had a broken hip in the past, which was fixed with treatment. The person can only walk short distances, has discomfort when moving around, and has some difficulty in daily activities.	heeft in het verleden de heup gebroken. De persoon kan alleen korte afstanden lopen, heeft een beetje pijn tijdens het bewegen en heeft wat moeite met het uitvoeren van dagelijkse activiteiten.
146	GBD 2013	Fracture other than femoral neck: short term, with or without treatment	has a broken thigh bone. The person has severe pain and swelling and cannot walk.	heeft een gebroken been. De persoon heeft ernstige pijn en zwellingen van het been en kan niet lopen.
147	GBD 2013	Fracture other than femoral neck: long term, without treatment	had a broken thigh bone in the past, which was never treated and did not heal properly. The person now has a limp and discomfort when walking.	heeft in het verleden een been gebroken. De persoon loopt nu mank en heeft wat pijn tijdens het lopen.
148	GBD 2010	Fracture of patella, tibia or fibula or ankle: short term, with or without treatment	has a broken shin bone, which causes severe pain, swelling, and difficulty walking.	heeft een gebroken scheenbeen. Deze persoon heeft veel pijn en zwelling van het onderbeen en heeft moeite met lopen.
149	GBD 2010	Fracture of patella, tibia or fibula or ankle: long term, with or without treatment	had a broken shin bone in the past that did not heal properly. The person has pain in the knee and ankle, and has difficulty walking.	heeft een gebroken scheenbeen gehad die niet volledig geheeld is. De persoon heeft pijn in de knie en enkel en heeft moeite met lopen.
150	GBD 2010	Fracture of pelvis: short term	has a broken pelvis bone, with swelling and bruising. The person has severe pain, and cannot walk or do daily activities.	heeft een gebroken bekken met zwelling en kneuzingen. De persoon heeft ernstige pijn en kan niet lopen of dagelijkse activiteiten uitvoeren.
151	GBD 2010	Fracture of pelvis: long term	had a broken pelvis in the past and now walks with a limp. There is often pain in the back and groin, and when urinating and sitting for a long time.	heeft in het verleden het bekken gebroken en loopt nu mank. De persoon heeft vaak pijn in de rug en lies tijdens het plassen en langdurig zitten.
152	GBD 2010	Fracture of radius or ulna: short term, with or without treatment	has a broken forearm, which causes severe pain, swelling, and limited movement.	heeft een gebroken onderarm. Deze persoon heeft ernstige pijn en zwelling van de onderarm en beperkte bewegingsvrijheid van de arm.
153	GBD 2010	Fracture of radius or ulna: long term, without treatment	had a broken forearm in the past that did not heal properly, causing some pain and limited movement in the elbow and wrist. The person has difficulty with daily activities such as dressing.	heeft in het verleden een gebroken arm gehad die niet volledig geheeld is. Dit leidt tot enige pijn en het minder goed kunnen bewegen van de elleboog en pols. De persoon heeft moeite met dagelijkse activiteiten, zoals aankleden.

154	GBD 2010	Fracture of skull: short or long term, with or without treatment	has a broken skull, but does not have brain damage. The broken area is painful and swollen	heeft schedelbreuk, maar heeft geen hersenletsel. Het aangedane deel van het hoofd is pijnlijk en opgezwollen.
155	GBD 2010	Fracture of sternum and/or fracture of one or two ribs: short term, with or without treatment	has a broken rib that causes severe pain in the chest, especially when breathing in. The person has difficulty with daily activities such as dressing.	heeft een gebroken rib. Deze persoon heeft ernstige pijn in de borst, met name tijdens het inademen. De persoon heeft moeite met dagelijkse activiteiten, zoals aankleden.
156	GBD 2010	Fracture of vertebral column: short or long term, with or without treatment	has broken back bones and is in pain, but still has full use of arms and legs.	heeft gebroken rug. Deze persoon heeft pijn aan de rug, maar kan armen en benen nog wel volledig gebruiken.
157	GBD 2010	Fractures, treated: long term	has slight pain in a bone that was broken in the past.	heeft een beetje pijn in een bot dat in het verleden gebroken is geweest.
158	GBD 2010	Injured nerves: short term	has a nerve injury, which causes difficulty moving and some loss of feeling in the affected area.	heeft een zenuwbeschadiging die leidt tot problemen met bewegen en verminderd gevoel in het aangedane lichaamsdeel.
159	GBD 2010	Injured nerves: long term	had a nerve injury in the past, which continues to cause some difficulty moving. The person often injures the affected part because it is numb.	heeft in het verleden een letsel aan de zenuwen gehad, dat nog steeds voor problemen tijdens het bewegen zorgt. De persoon heeft geen gevoel meer in dit deel van het lichaam en loopt daardoor vaak nieuwe verwondingen op.
160	GBD 2010	Injury to eyes: short term	has an injury to one eye, which causes pain and difficulty seeing.	heeft een verwonding aan een oog, wat leidt tot pijn en moeite met zien.
161	GBD 2013	Concussion	has headaches, dizziness, nausea and difficulty concentrating.	heeft hoofdpijn en is duizelig en misselijk. Deze persoon heeft ook moeite met concentreren.
162	GBD 2010	Traumatic brain injury: severe, short term, with or without treatment	cannot concentrate and has headaches, memory problems, dizziness, and feels angry.	kan zich niet concentreren en heeft hoofdpijn, geheugenproblemen, duizeligheid en voelt zich boos.
163	GBD 2010	Traumatic brain injury, long-term consequences, minor, with or without treatment	has episodes of headaches, memory problems, and difficulty concentrating.	heeft last van hoofdpijn, geheugenproblemen en concentratieproblemen.
164	GBD 2010	Traumatic brain injury, long-term consequences, moderate, with or without treatment	has frequent headaches, memory problems, difficulty concentrating, and dizziness. The person is often anxious and moody.	heeft regelmatig hoofdpijn, geheugenproblemen, concentratieproblemen en duizeligheid. De persoon is vaak nerveus en humeurig.
165	GBD 2010	Traumatic brain injury, long-term consequences, severe, with or without treatment	cannot think clearly and has frequent headaches, memory problems, difficulty concentrating and dizziness. The person is often anxious and moody, and depends on others for feeding, toileting, dressing and walking.	kan niet helder denken en heeft regelmatig hoofdpijn, geheugenproblemen, concentratieproblemen en duizeligheid. De persoon is vaak nerveus en humeurig en heeft anderen nodig om te eten, naar het toilet te gaan, voor het aankleden en lopen.
166	GBD 2010	Open wound: short term, with or without treatment	has a cut in the skin, which causes pain and numbness around the cut	heeft een snee in de huid die leidt tot pijn rond de wond.
167	GBD 2010	Poisoning: short term with or without treatment	has drowsiness, stomach pain and vomiting.	voelt zich duizelig, heeft last van maagpijn en overgeven.

168	GBD 2010	Severe chest injury: short term, with or without treatment	has a serious chest injury, which causes severe pain, shortness of breath and anxiety.	heeft een ernstige verwonding aan de borstkas, die leidt tot ernstige pijn, kortademigheid en angst.
169	GBD 2010	Severe chest injury: long term, with or without treatment	had a severe chest injury in the past that has now healed. The person still gets breathless when walking and feels discomfort in the chest.	heeft in het verleden een ernstige verwonding aan de borstkas gehad die nu geheeld is. De persoon raakt nog steeds buiten adem tijdens het lopen en voelt wat ongemak in de borstkas.
170	GBD 2013	Spinal cord lesion below neck level: treated	is paralyzed from the waist down, cannot feel or move the legs and has difficulties with urine and bowel control. The person uses a wheelchair to move around.	is vanaf de middel verlamd, kan de benen niet voelen of bewegen en weinig controle over urine en darmen en is daardoor soms incontinent. De persoon gebruikt een rolstoel om te bewegen.
171	GBD 2013	Spinal cord lesion below neck level: untreated	is paralyzed from the waist down, cannot feel or move the legs and has difficulties with urine and bowel control. Legs are in fixed, bent positions, and the person gets frequent infections and pressure sores.	is vanaf de middel verlamd, kan de benen niet voelen of bewegen en heeft weinig controle over urine en darmen en is daardoor soms incontinent. De persoon heeft regelmatig last van infecties en drukwonden aan de benen.
172	GBD 2013	Spinal cord lesion at neck level: treated	is paralyzed from the neck down, with no feeling or control over any part of the body below the neck, and no urine or bowel control.	is vanaf de nek verlamd en heeft geen gevoel in of controle over het lichaam onder de nek. Deze persoon heeft geen controle over urine of darmen en is daardoor incontinent.
173	GBD 2013	Spinal cord lesion at neck level: untreated	is paralyzed from the neck down, with no feeling or control over any part of the body below the neck, and no urine or bowel control. Arms and legs are in fixed, bent positions, and the person gets frequent infections and pressure sores.	is vanaf de nek verlamd en heeft geen gevoel in of controle over een deel van het lichaam onder de nek, zoals armen, rug en benen. Deze persoon heeft geen controle over urine of darmen en is daardoor incontinent en de persoon heeft regelmatig last van infecties en drukwonden.
174	*New*	Injury to internal organs	has a lot of pain in the abdomen and is occasionally unconscious.	heeft veel pijn ter hoogte van de buik en is af en toe buiten bewustzijn.
175	*New*	Broken teeth	has broken teeth. This person has a lot of pain in his teeth.	heeft afgebroken tanden. Deze persoon heeft veel pijn aan de tanden.
176	*New*	Broken nose	has a broken nose. The nose is swollen and very painful.	heeft een gebroken neus. De neus is gezwollen en heel pijnlijk.
177	*New*	Broken cheekbone	has a broken cheekbone, with swelling and severe pain.	heeft een gebroken jukbeen, met zwellingen en ernstige pijn.
		**Other**		
178	*New*	Annoyance: moderate	is continuously moderately annoyed because of loud noises in the surroundings, is irritable and has some difficulty concentrating.	is doorlopend geïrriteerd door harde omgevingsgeluiden, is prikkelbaar en heeft een beetje moeite met concentreren.

179	*New*	Annoyance: severe	is continuously highly annoyed because of loud noises in the surroundings, is irritable and has difficulty concentrating.	is doorlopend zeer geïrriteerd door harde omgevingsgeluiden, is erg prikkelbaar en heeft moeite met concentreren.
180	*New*	Cognitive impairments: mild	is slow in learning and has difficulty doing complex or unfamiliar tasks but otherwise functions independently.	leert langzaam en heeft moeite met het uitvoeren van ingewikkelde taken, maar functioneert verder zelfstandig.
181	*New*	Cognitive impairments: moderate	is slow in learning to speak and to do simple tasks. As an adult, the person requires support to live independently and raise children and can only work at simple supervised jobs.	heeft moeite met leren praten en het uitvoeren van eenvoudige taken. Als volwassene heeft de persoon veel hulp nodig in het dagelijkse leven. Deze persoon kan alleen werken in eenvoudige banen onder toezicht.
182	*New*	Cognitive impairments: severe	is slow in learning to speak and to do simple tasks. As an adult, the person needs constant supervision and help with all daily activities, like washing and dressing.	heeft veel moeite met leren praten en het uitvoeren van eenvoudige taken. Deze persoon heeft doorlopend toezicht nodig en hulp bij alle dagelijkse activiteiten en zelfzorg, zoals wassen en aankleden.
183	*New*	Constitutional eczema: mild/moderate	has red and scaly patches of skin and itching, but has no problems with daily activities.	heeft rode en schilferige plekken op de huid en jeuk, maar heeft geen problemen met dagelijkse activiteiten.
184	*New*	Constitutional eczema: severe	has large red and scaly patches of skin all over the body and intense itching. This person has worries and fears and has some difficulty performing daily activities.	heeft op het hele lichaam grote rode en schilferige plekken op de huid en hevige jeuk. Deze persoon heeft zorgen en angsten en heeft wat problemen met het uitvoeren van dagelijkse activiteiten.
185	*New*	Fatigue	often has little energy, which causes many problems while carrying out daily activities.	heeft meestal weinig energie, wat zorgt voor veel problemen tijdens het uitvoeren van de dagelijkse activiteiten.
186	*New*	Impaired self-care	has difficulty with self-care, like washing and dressing.	heeft moeite met zelfzorg, zoals wassen en aankleden.
187	EURO	Intensive care unit admission	is very ill and often asleep or unconscious; when awake cannot move in bed, cannot speak, is completely dependent on others and is anxious.	is erg ziek en vaak buiten bewustzijn. Deze persoon kan niet bewegen of spreken, is volledig afhankelijk van anderen en is angstig.
188	*New*	Loneliness	feels disconnected from other people. This person has less close contact with others than desired.	voelt zich niet verbonden met andere mensen. Deze persoon heeft een minder hecht contact met anderen dan gewenst.
189	GBD 2010	Motor impairment: mild	has some difficulty in moving around but is able to walk without help.	heeft een beetje moeite met bewegen, maar kan zonder hulp lopen.
190	GBD 2010	Motor impairment: moderate	has some difficulty in moving around, and difficulty in lifting and holding objects, dressing and sitting upright, but is able to walk without help.	heeft enige moeite met het bewegen, het vasthouden van dingen, aankleden en rechtop zitten, maar kan zonder hulp lopen.

191	GBD 2010	Motor impairment: severe	is unable to move around without help, and is not able to lift or hold objects, get dressed or sit upright.	kan niet zonder hulp rondlopen en kan geen objecten optillen of vasthouden, zich aankleden of rechtop zitten.
192	EURO	Motor and cognitive impairments: mild	has some difficulty in moving around but is able to walk without help. The person is slow in learning at school. As an adult, the person has some difficulty doing complex or unfamiliar tasks but otherwise functions independently.	heeft wat moeite met bewegen, maar kan zonder hulp lopen. Deze persoon leert langzaam op school. Als volwassene heeft de persoon wat moeite met het uitvoeren van complexe of onbekende taken, maar functioneert verder onafhankelijk.
193	EURO	Motor and cognitive impairments: moderate	has some difficulty in moving around, holding objects, dressing and sitting upright, but can walk without help. The person has low intelligence and is slow in learning to speak and to do simple tasks. As an adult, the person requires support to live independently and raise children and can only work at simple supervised jobs.	heeft wat moeite met het bewegen, het vasthouden van dingen, aankleden en rechtop zitten, maar kan zonder hulp lopen. Deze persoon heeft een lage intelligentie en is langzaam in het leren praten en het uitvoeren van eenvoudige taken. Als volwassene heeft de persoon hulp nodig om onafhankelijk te leven en kinderen op te voeden en kan deze alleen werken in eenvoudige banen onder toezicht.
194	EURO	Motor and cognitive impairments: severe	cannot move around without help, and cannot lift or hold objects, get dressed or sit upright. The person also has very low intelligence, speaks few words, and needs constant supervision and help with all daily activities.	kan niet zonder hulp rondlopen en kan dingen niet optillen of vasthouden, zich aankleden of rechtop zitten. De persoon heeft ook een lage intelligentie, spreekt een paar woorden en heeft doorlopend toezicht nodig en hulp bij alle dagelijkse activiteiten.
195	*New*	Sleep disturbance	has difficulty falling or staying asleep. As a result, this person is tired and has some problems performing daily activities.	heeft moeite met in slaap te vallen of te blijven. Hierdoor is deze persoon moe en heeft enkele problemen met het uitvoeren van dagelijkse activiteiten.
196	*New*	Vertigo and balance disorder (Menière, labyrinthitis)	suffers from dizziness, light-headedness and is anxious.	heeft last van draaierigheid, een licht gevoel in het hoofd en is angstig.
197	*New*	Generic, mild health problems	at times has low energy but is able to perform daily activities.	heeft soms weinig energie, maar kan dagelijkse activiteiten uitvoeren.
198	*New*	Generic, mild physical and mental health problems	has low energy some of the time, has some aches and at times feels downhearted, but is able to perform daily activities.	heeft soms weinig energie en wat pijn en voelt zich soms verdrietig, maar kan de dagelijkse activiteiten uitvoeren.
199	*New*	Generic, mild physical and mental health problems and mild functional limitations	has low energy some of the time, has some difficulty moving around, has moderate pain and at times feels downhearted, but is able to perform daily activities.	heeft soms weinig energie, wat moeite met bewegen, matige pijn en voelt zich soms verdrietig, maar kan de dagelijkse activiteiten uitvoeren.
200	*New*	Generic, mild mental health problems and moderate physical health problems and functional limitations	has low energy some of the time, has some difficulty in moving around, has moderate pain and sometimes feels downhearted, causing difficulty with daily activities.	heeft soms weinig energie, wat moeite met bewegen, matige pijn en voelt zich soms verdrietig, wat zorgt voor problemen tijdens het uitvoeren van de dagelijkse activiteiten.

201	*New*	Generic, moderate mental health problems and severe physical health problems and functional limitations	has low energy most of the time, has great difficulty in moving around, has a lot of pain and sometimes feels downhearted, causing great difficulty with daily activities.	heeft meestal weinig energie, veel moeite met bewegen, veel pijn en voelt zich verdrietig, wat zorgt voor veel problemen tijdens het uitvoeren van de dagelijkse activiteiten.
202	*New*	Generic, severe physical and mental health problems and severe functional limitations	has low energy most of the time, has great difficulty in moving around, has a lot of pain and feels downhearted most of the time, causing great difficulty with daily activities.	heeft meestal weinig energie, veel moeite met bewegen, veel pijn en voelt zich meestal verdrietig, wat zorgt voor veel problemen tijdens het uitvoeren van de dagelijkse activiteiten.
203	*New*	Generic, extreme physical and mental health problems and extreme functional limitations	has low energy all of the time, has great difficulty in moving around, has extreme pain and feels downhearted all of the time, making it impossible to carry out daily activities.	heeft altijd weinig energie, veel moeite met bewegen, extreme pijn en voelt zich altijd verdrietig, wat het uitvoeren van de dagelijkse activiteiten onmogelijk maakt.

EURO: European disability weights measurement study; GBD: Global Burden of Disease; IQ: Intelligence Quotient
Note: Brief lay descriptions (in English and Dutch) of the seven experimental health states are available from the corresponding author upon request.

2.2. Table 2: The Netherlands Sub-regions

The Netherlands is divided into four **NUTS1 regions**, each with distinct sociodemographic characteristics.

1. Northern Netherlands: This region covers the three Northern provinces of Groningen, Friesland, and Drenthe. Compared to the other NUTS1 regions it has a lower population density and a higher proportion of rural areas. The population in this region is slightly older, with a relatively high number of elderly residents.
2. Eastern Netherlands: This region includes the provinces of Overijssel, Gelderland, and Flevoland. This region encompasses urban and rural areas. The population is relatively balanced in terms of age distribution.
3. Southern Netherlands: This region encompasses the provinces of North-Brabant and Limburg. The population has a balanced age distribution and has a mix of urban and rural communities.
4. Western Netherlands: This region includes the provinces of North Holland, South Holland, Utrecht, and Zeeland and is the most urbanized and densely populated region. It contains major cities like Amsterdam, Rotterdam, The Hague, and Utrecht. The population tends to be younger compared to the other regions.

**Sub-region**	**Province or municipality**
North	Groningen, Friesland, Drenthe
East	Overijssel, Gelderland, Flevoland
South	Noord-Brabant, Limburg
West	Utrecht, Noord-Holland, Zuid-Holland, Zeeland

2.3. Figure 1: Age-gender distribution of the Dutch Disability Weight cohort sample *versus* the national population

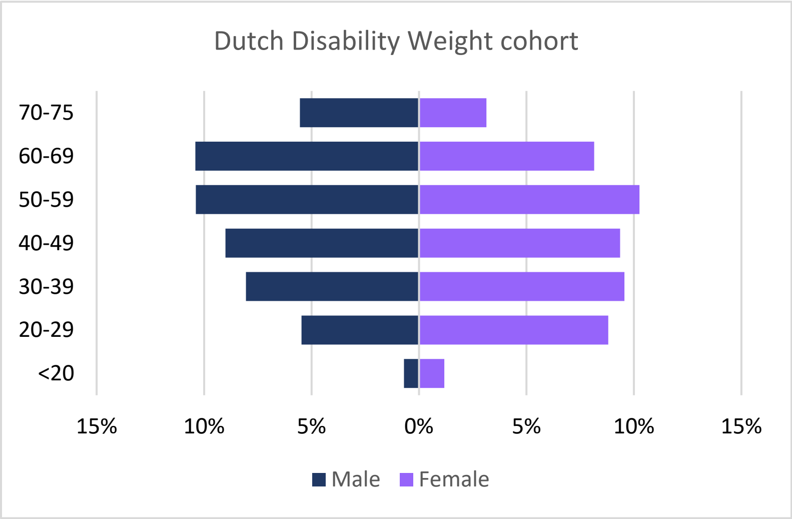

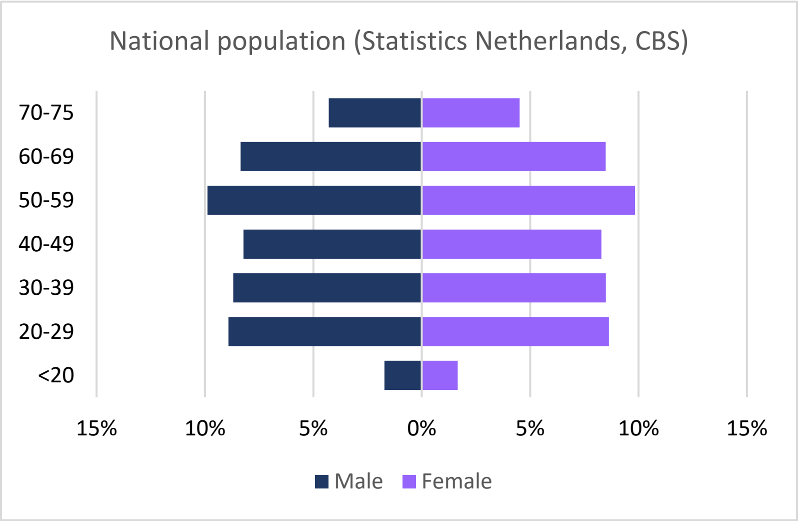

2.4. Figure 2: Age-gender-education distribution of the Dutch Disability Weight cohort sample, by regions

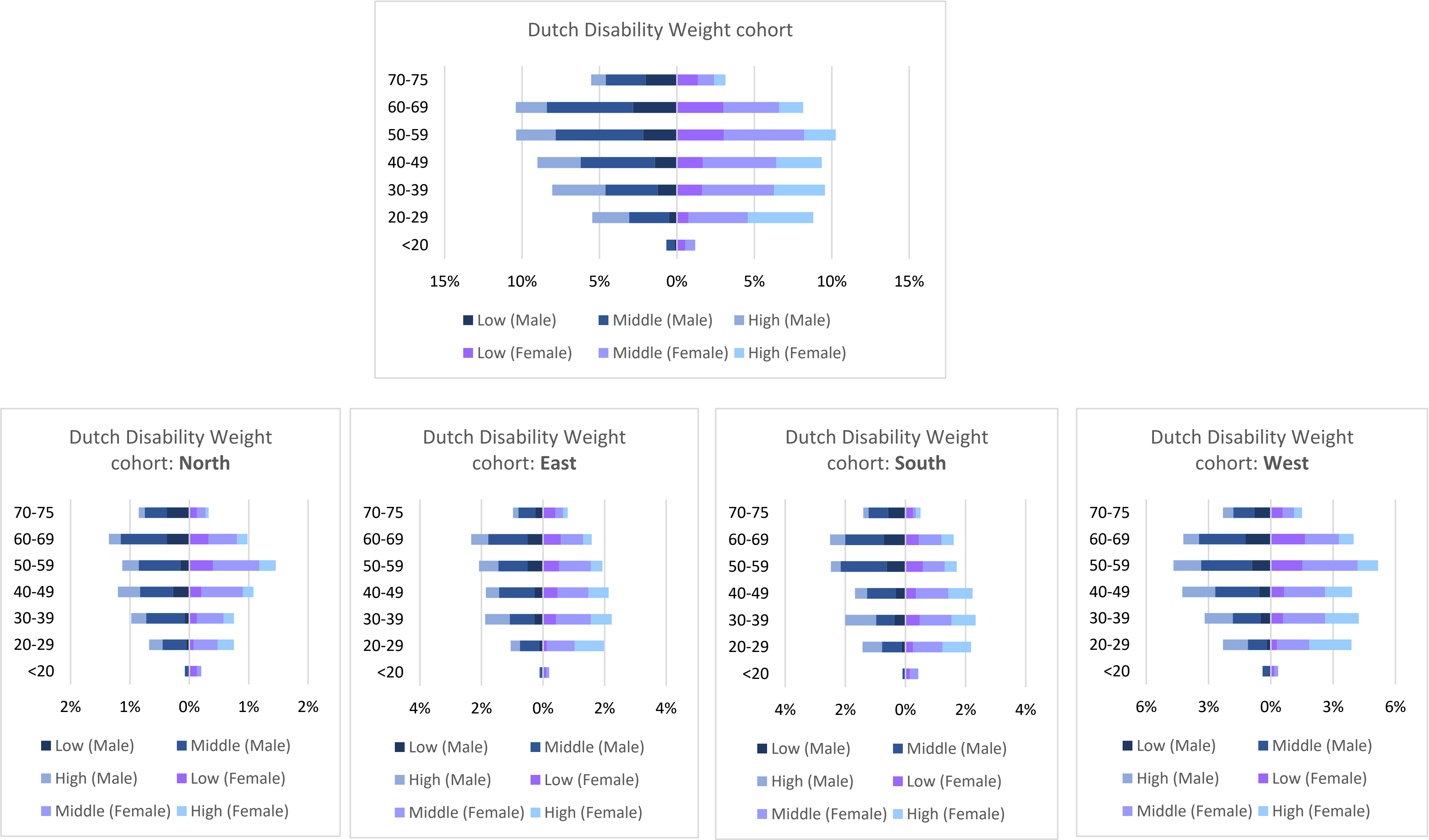

2.5. Test re-test analysis of the paired comparison, by NL-pooled and NL-region and educational level

All participants were given the same pair of health states in the 2nd and 15th paired comparison questions, and of these 48.1% were presented in same order, and the remaining 51.9% were presented in reversed order. This deliberate repetition allows assessment of reliability of paired comparison question using a test re-test analysis.

Table 3: Test re-test analysis of the paired comparison, by NL-pooled and NL-region and educational level

	**Netherlands Sub-region**				**Pooled**
	**North**	**East**	**South**	**West**	
**Overall**	0.738	0.730	0.738	0.732	0.734
Same order	0.785	0.728	0.748	0.732	0.741
Reverse order	0.699	0.732	0.727	0.731	0.727
**Low educated**	0.645	0.731	0.744	0.736	0.727
Same order	0.654	0.744	0.778	0.751	0.745
Reverse order	0.638	0.732	0.709	0.723	0.711
**Middle educated**	0.755	0.733	0.719	0.720	0.727
Same order	0.813	0.737	0.723	0.730	0.740
Reversed order	0.703	0.729	0.716	0.711	0.714
**High educated**	0.794	0.719	0.764	0.746	0.749
Same order	0.872	0.702	0.770	0.721	0.739
Reverse order	0.746	0.739	0.759	0.768	0.758

Note: White-to-light blue cells correspond to response probabilities between 0.638 and 0.741. Blue cells correspond to response probabilities between 0.742 and 0.785. Purple cells correspond to response probabilities greater than 0.785.

2.6. Figure 4: Spearman's correlation of probit coefficients by age, gender, highest attained level of education, chronic disease status, and region of residence

Socio-demographic attribute	Dutch Disability Weight cohort
**Age (in years)**	
18-34 *versus* 35-54	0.956*
18-34 *versus* 55-75	0.962*
35-54 *versus* 55-75	0.978*
**Education level**	
Low *versus* Middle	0.970*
Low *versus* High	0.953*
Middle *versus* High	0.974*
**Gender**	
Male *versus* Female	0.978*
**Chronic disease status**	
Yes *versus* No	0.980*
**Region of residence**	
North *versus* East	0.931*
North *versus* South	0.946*
North *versus* West	0.954*
East *versus* South	0.955*
East *versus* West	0.964*
South *versus* West	0.966*

* Correlation is significant at the 0.01 level.

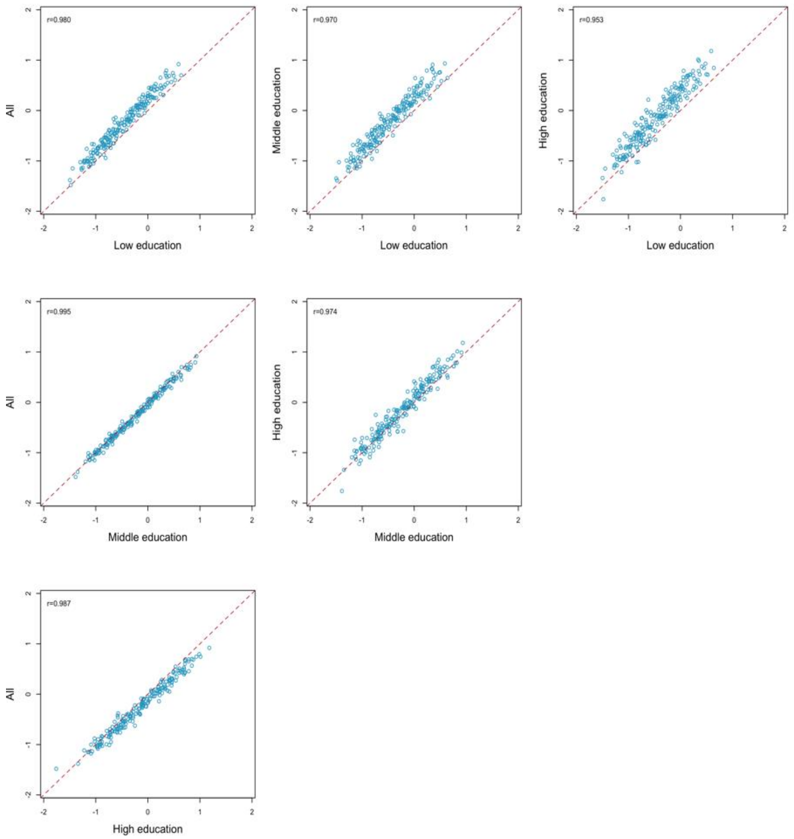

2.6. Figure 4: Spearman's correlation of probit coefficients by age, gender, highest attained level of education, chronic disease status, and region of residence *(continued from previous page)*

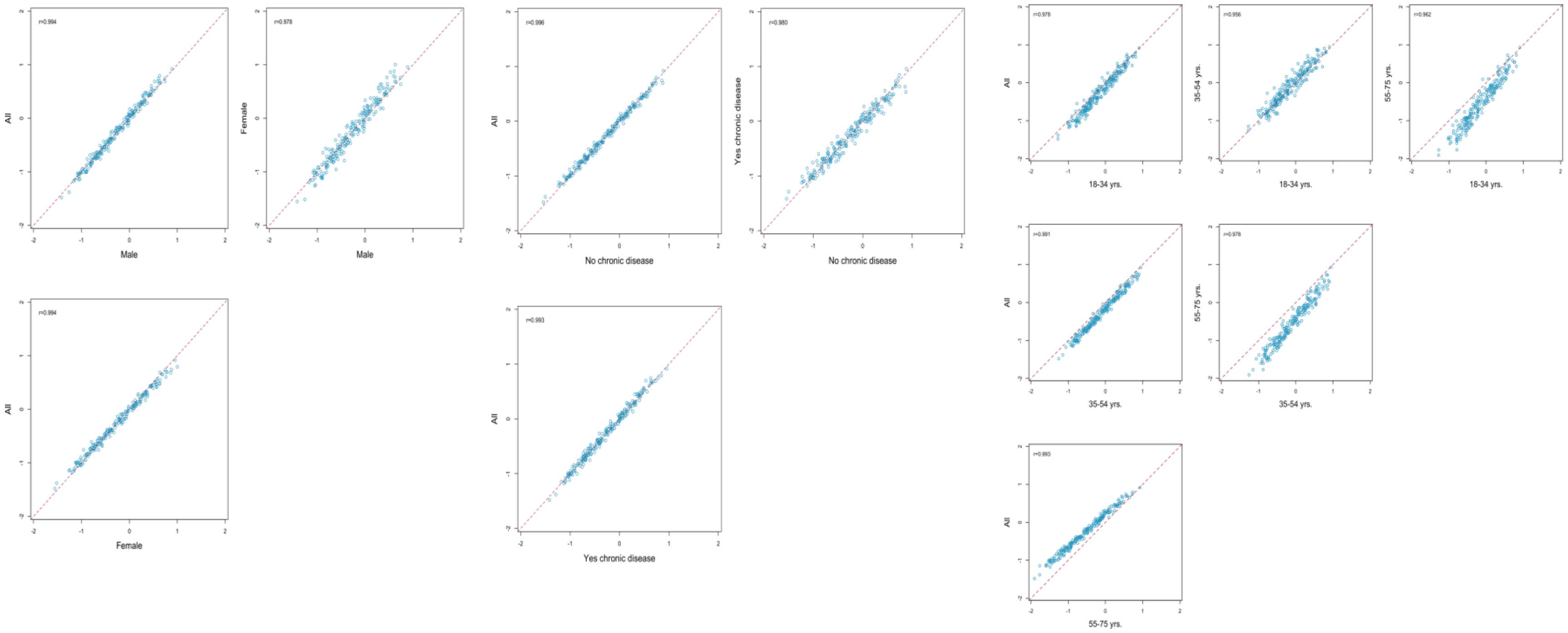

2.6. Figure 4: Spearman's correlation of probit coefficients by age, gender, highest attained level of education, chronic disease status, and region of residence *(continued from previous page)*

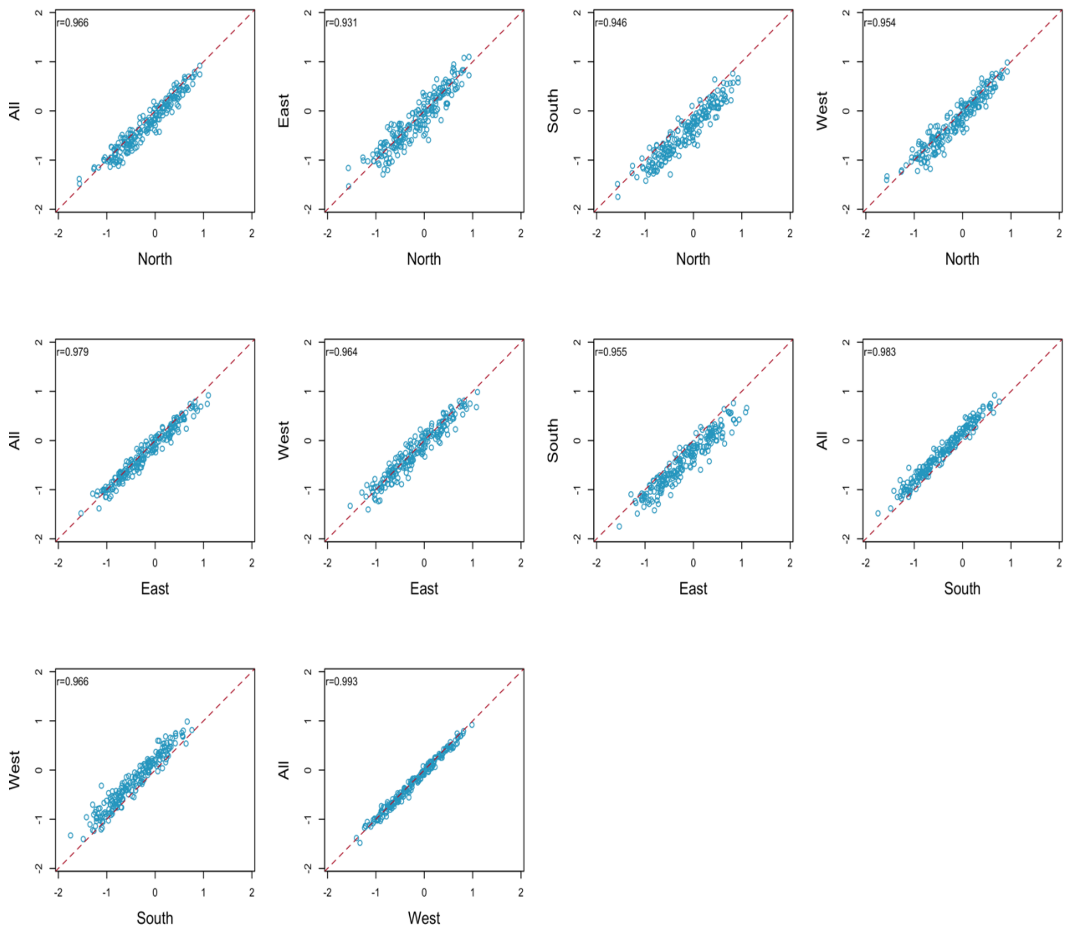

2.7. Table 4: Comparison of Dutch and European disability weights

Comparison of the disability weights of the 140 health states that were included in both the Dutch and European disability weights study showed a high correlation (Spearman's correlation: 0.942; p<0.001). The median disability weight of all 140 health states did not differ significantly between the Dutch and the European disability weight study (median of Dutch disability weights: 0.103, interquartile range (IQR) 0.041-0.291; median of European disability weights: 0.122, IQR 0.044-0.281); p=0.972). However, for 76 (54.3%) of the 140 health states the point estimate of the Dutch disability weight fell outside of the 95%UI of the European disability weights. For 33 (23.6%) health states, the Dutch disability weights were lower than the lower bound. Approximately one in three of the neurological and injury health states was significantly lower than the European disability weights. For 43 (30.7%) health states, the Dutch disability weights were higher than the higher bound of the 95%UI of the European disability weights. All of the diabetes, digestive, and genitourinary disease health states and four in five cardiovascular and circulatory diseases were significantly higher than the European disability weights.

	**Number of health states in both studies**	**Health states significantly lower or higher compared to the European disability weights measurement study**			
		Lower		Higher	
**Disease category**	N	*n*	*%*	*n*	*%*
Infectious diseases	7	1	14.3	4	57.1
Cancer	5	2	40.0	2	40.0
Cardiovascular and circulatory diseases	5	0	0.0	4	80.0
Diabetes, digestive, and genitourinary disease	5	0	0.0	5	100.0
Respiratory diseases	5	0	0.0	3	60.0
Neurological disorders	10	3	30.0	4	40.0
Mental, behavioural, and substance use disorders	12	3	25.0	5	41.7
Hearing and vision loss	14	3	21.4	1	7.1
Musculoskeletal disorders	11	0	0.0	3	27.3
Injury	57	17	29.8	10	17.5
Other	9	4	44.4	2	22.2
Total	140	33	23.6	43	30.7
