# Supplementary material for: Deriving disability weights for the Netherlands: findings from the Dutch disability weights measurement study
Source: Popul Health Metr. 2024 Oct 7;22:26. doi: 10.1186/s12963-024-00342-0 (PMC11457395; doi:10.1186/s12963-024-00342-0)
Supplement: Supplementary file 2 — Supplementary Material 2 [file 12963_2024_342_MOESM2_ESM.pdf]

Dutch Disability Weights: R code on paired comparison analyses

Periklis Charalampous & Carolien C.H.M. Maas

Task 1: Paired comparison response probabilities:heatmap

```r
# create dummy when choosing first person
data$choosefirst <- as.numeric(data$answer_matrix==1)

# function that maps probabilities to heat colors (i.e., returns matrix)
color.map <- function(heat.mat, rgb.color=color){
  # bin the heat.mat values into the subintervals
  heat.values = as.vector(heat.mat)
  binned = cut(x=heat.values, breaks=seq(from=0, to=1, by=1/length(rgb.color)),
               right=T, include.lowest=T, labels=rgb.color)
  bin.mat = matrix(binned, nrow=nrow(heat.mat), byrow = F,
                   dimnames=list(rownames(heat.mat), colnames(heat.mat)))
  return(bin.mat)
}

# rgb colors for images
red <- c(rep(255, 256), seq(254, 0, -1), rep(0, 255), rep(0, 255))
green <- c(seq(0, 255, 1), rep(255, 255), rep(255, 255), seq(254, 0, -1))
blue <- c(rep(0, 256), rep(0, 255), seq(1, 255, 1), rep(255, 255))
color <- rgb(red = red, green = green, blue = blue, maxColorValue = 255)

# create probability of choosing first person
# for each combination of description_first_person and
# description_second_person in the data set
summarized.data <- data %>%
  group_by(description_first_person, description_second_person) %>%
  summarise(prob=mean(choosefirst))

# create matrix probabilities
nr.health.states <- 210
matrix.prob <- matrix(NA, nrow=nr.health.states, ncol=nr.health.states)
for (A in sort(unique(as.numeric(as.character(data$description_first_person))))){
for (B in sort(unique(as.numeric(as.character(data$description_first_person))))){
    matrix.prob[A+1, B+1] <- as.numeric(subset(summarized.data,
                                              description_first_person==A &
                                                description_second_person==B)
                                       [, c("prob")])
  }
}
```

```r
# order the probability matrix
matrix.prob <- matrix.prob[order(rowMeans(matrix.prob, na.rm=TRUE),
                                decreasing=TRUE),
                          order(colMeans(matrix.prob, na.rm=TRUE))]

# parameter that controls the size of the color gradient legend (optional)
# note:  it must be odd, decrease n to make legend larger/
#        increase n to make it smaller
n <- 13

# converts data frame into a matrix
heatProbs <- data.matrix(frame=matrix.prob)
heatMatrix <- color.map(heat.mat=heatProbs, rgb.color=color) #matrix_heat_colors

# open figure
png(file=paste0(file.path, "/Results/Figures/heatmap.png"),
    width=16, height=16, units="cm", res=300)

# plot an empty initial graph
par(mar=rep(2, 4))
plot(x = c(), y = c(), xlim = c(0,1), ylim = c(0,1), xlab = "",
     ylab = "", axes = F, main="", cex.main=2)

# assign plot parameters
x.a <- par()$usr[1];  x.b <- par()$usr[2];
y.a <- par()$usr[3];  y.b <- par()$usr[4];

# calculate step sizes
h.x <- (x.b - x.a) / nrow(heatMatrix)
h.y <- (y.b - y.a) / ncol(heatMatrix)

# mesh coordinates of heat maps
x.s <- x.a + (0:nrow(heatMatrix)) * h.x
y.s <- y.a + (0:ncol(heatMatrix)) * h.y

# plotting the heat colors to respective rectangles
for (sims2 in 1:nrow(heatMatrix)){
  rect(xleft = rep(x.s[sims2], ncol(heatMatrix)),
       ybottom = y.s[-length(y.s)],
       xright = rep(x.s[sims2+1], ncol(heatMatrix)),
       ytop = y.s[-1], col = heatMatrix[sims2,], border = NA)
}

# add box
box()

#close figure
grDevices::dev.off()
```

```
## pdf
##   2
```

```r
#Plot color gradient legend
png(file=paste0(file.path, "/Results/Figures/heatmap_legend.png"),
    width=4, height=16, units="cm", res=300)
par(mar=c(2, 0, 2, 0))
plot(x = c(), y = c(), xlim = c(0,1), ylim = c(0,1), xlab = "",
     ylab = "", axes = F)
xmid <- median(1:n)
x <- seq(from=x.a, to=x.b, by=(x.b - x.a)/n)
y <- seq(from=y.a, to=y.b, by=(y.b - y.a)/length(color))

# creates colorful legend
rect(xleft=x[xmid], xright=x[xmid+1], ybottom=y[-length(y)],
     ytop=y[-1], col=color, border=NA)

# define middle
par1 <- (x[xmid+1] + x[xmid+2])/2

# makes the verticle line
abline(v=par1, col="black")
probs <- cut(x=seq(0,0.75,by=0.25), breaks=seq(from=0, to=1, by=1/length(color)),
             right=T, include.lowest=T)

# creates horizontal stripes
segments(x0=par1, x1=((x[2]-x[1])/4) + par1,
         y0=y[c(as.numeric(probs), length(y))],
         y1=y[c(as.numeric(probs), length(y))])

# adds text
text(x=((x[2]-x[1])/2) + par1, y=c(-0.02, y[(as.numeric(probs))[-1]], 1.03),
     label = c(0, 0.25, 0.5, 0.75, 1), cex=0.75, pos=4)

# close figure
grDevices::dev.off()
```

```
## pdf
##   2
```

```r
knitr::include_graphics(paste0(file.path, "/Results/Figures/heatmap.png"))
```

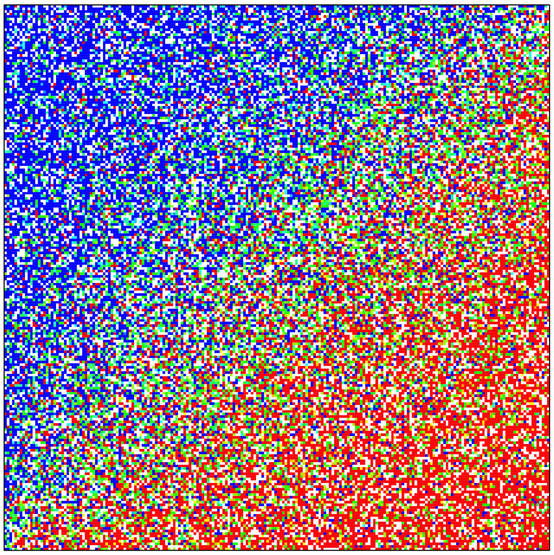

Task 2: Perform probit analysis:estimation of DDW

```
# create empty dummies
nr.health.states <- 210
list.state.nrs <- sort(unique
                       (as.numeric(as.character(data$description_first_person))))
data[, paste0('picked', list.state.nrs)] <- rep(0, nrow(data))

# create indicator variables that indicate
# 1 if that health state is chosen as the healthier
# -1 if the health state is not chosen as the healthier
# 0 if that health state was not considered
for (state in list.state.nrs){
data[which(data$description_first_person==state), paste0('picked', state)] <- 1
data[which(data$description_second_person==state), paste0('picked', state)] <- -1
}
data <- data.frame(data)

# probit regression
formula <- paste("choosefirst ~ -1 + ",
                 paste(names(data)[12:221], collapse=" + "),
                 sep="")
print(formula)
```

```
## [1] "choosefirst ~ -1 + picked0 + picked1 + picked2 + picked3 + picked4 + picked5 + picked6 + picked
```

```
output_All <- stats::glm(formula, family = binomial(link="probit"), data=data)
```

```r
# picked209 is the reference category
coef_All <- summary(output_All)$coefficients[, 1]
openxlsx::write.xlsx(data.frame(Names=names(coef_All),
                                Coefficients=coef_All),
                     rowNames=FALSE,
                     file=paste0(file.path, "/Results/Coefficients_PC.xlsx"))

# define logit and expit functions to calculate disability weights
logit <- function(x) log(x / (1 - x))
expit <- function(x) exp(x) / (1 + exp(x))

## GBD data
GBD <- c (0.074, NA, 0.408, 0.078, 0.274, 0.582, 0.006, 0.051, 0.133, 0.288,
          0.036, 0.451, 0.095,0.54, NA, 0.569, NA, 0.133, 0.02, 0.069, 0.377,
          0.449, 0.235, 0.57, 0.778, 0.588, 0.145, 0.396, 0.658, 0.03, 0.133,
          0.523, NA, NA, NA, NA, NA, NA, 0.043, 0.1, 0.16, 0.2, NA, NA, NA,
          NA, NA, 0.01, 0.267, 0.575, 0.183, 0.463, 0.719, 0.263, NA, 0.037,
          NA, 0.441, 0.019, 0.07, 0.552, 0.003, 0.031, 0.184, 0.187, 0.011,
          0.017, 0.041, 0.072, 0.179, 0.432, 0.224, NA, 0.019, 0.225, 0.408,
          0.015, 0.036, 0.133, NA, NA, NA, 0.028, 0.317, 0.581, NA, NA, 0.295,
          0.053, 0.114, 0.229, 0.02, 0.054, 0.372, NA, NA, 0.571, NA, NA,
          NA, NA, 0.492, 0.032, NA, NA, 0.088, 0.443, 0.123, NA, 0.005, 0.039,
          0.173, 0.118, NA, 0.011, 0.006, 0.016, 0.141, 0.314, 0.135, 0.455,
          0.11, 0.132, 0.016, 0.113, 0.062, NA, 0.247, 0.035, 0.026, 0.026,
          0.014, 0.01, 0.058, 0.258, 0.055, 0.05, 0.182, 0.279, 0.043, 0.028,
          0.071, 0.103, 0.111, 0.111, 0.042, 0.005, NA, NA, NA, 0.215, 0.316,
          0.01, 0.021, 0.027, 0.074, 0.204, 0.277, 0.158, 0.261, 0.113, 0.1,
          0.054, NA, 0.031, 0.203, 0.542, NA, 0.006, NA, 0.163, 0.047, 0.369,
          0.214, NA, NA, 0.296, NA, NA, 0.231, 0.637, NA, NA, 0.01, 0.061,
          0.402, NA, NA, NA, NA, NA, NA, NA, NA, NA, NA, NA, NA, NA, NA, NA,
          0.02, NA, NA, 0.165, 0.079, 0.117, NA, NA, NA)

# select coefficients
coef_All <- c(coef(output_All)[-nr.health.states], 0) #repl. coef of ref. by 0
names(coef_All) <- paste0('picked', list.state.nrs)

# run a non-parametric regression model (loess) of the probit regression
# coefficients against the logit-transformed disability weights from GBD
fit_loess <- loess(logit(GBD) ~ coef_All,
                     control=loess.control(surface="direct"))

# predicted logit transformed disability weights for each of the probit coef
pred_GBD <- predict(fit_loess, coef_All, se = TRUE)

# plot of LOESS regression
plot(logit(GBD) ~ coef_All, xlab=paste0("Coefficients of pooled"))
test <- data.frame(x=coef_All, y=pred_GBD$fit)
ordered <- test[order(test$x),]
lines(ordered$x, ordered$y, col="blue")
```

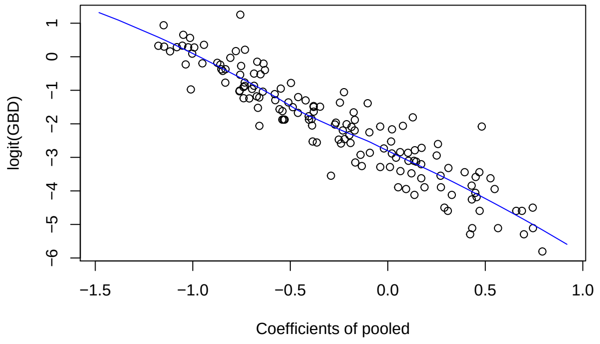


```
# calculate R-square
ss.resid <- sum(fit_loess$residuals^2)
ss.dist <- sum((logit(GBD)-mean(logit(GBD), na.rm=TRUE))^2, na.rm=TRUE)
Rsquared_GBD_All <- 1-ss.resid/ss.dist

# calculate disability weights using bootstrapping
coef_se <- summary(output_All)$coefficients[, c("Estimate", "Std. Error")]
n_samples <- 1000

# sample coefficients
coef_sim <- apply(coef_se, 1, function(x) rnorm(n_samples, x[1], x[2]))



# perform loess regression on old vs new coefficients for each sampled
dw_sim <-
  apply(coef_sim,
        1,
        function(x) {
          fit_loess <- loess(logit(GBD) ~ c(x, 0),
                             control=loess.control(surface="direct"))
          pred <- predict(fit_loess, c(x, 0), se = TRUE)
          dw_sim <- mapply(rnorm,
                           mean = pred$fit, sd = pred$se.fit,
                           MoreArgs = list(n = n_samples))
        })
dw_sim <- expit(dw_sim)

# calculate mean and CI of disability weights
summarize <-
  function(x, round = 3) {
    m <- mean(x, na.rm = TRUE)
    ci <- quantile(x, c(.025, .975), na.rm = TRUE)
    n_na <- sum(is.na(x))
    if (n_na > 0) warning("there were", n_na, "NA")
    return(round(c(mean = m, ci), round))
  }
```

```r
# mean disbability weights using numerical integration
out <- matrix(ncol = 3, nrow = nr.health.states)
for (i in seq(nr.health.states)) {
  out[i, ] <- summarize(dw_sim[(((i - 1) * n_samples) + 1):(i * n_samples), ])
}

# store results
DW_table <- data.frame(names=paste0('picked', list.state.nrs))
DW_table[, "DW_GBD_All"] <- out[,1]
DW_table[, "CI_lower_GBD_All"] <- out[,2]
DW_table[, "CI_upper_GBD_All"] <- out[,3]

# applied an inverse logit transformation at the draw level to predicted dw
plot(expit(pred_GBD$fit), coef_All)
```

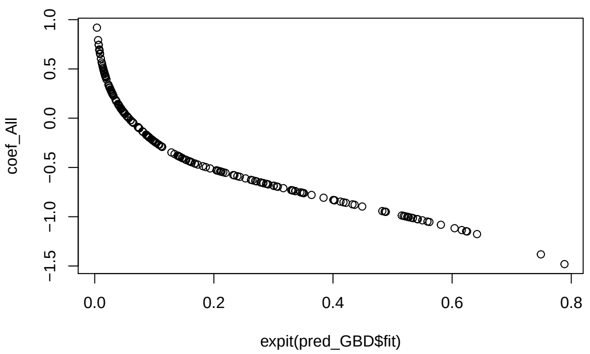


```r
# check that bootstrapped DW are similar to DW from probit analysis
round(expit(pred_GBD$fit)-DW_table[, "DW_GBD_All"], 3)
```

```
##   picked0   picked1   picked2   picked3   picked4   picked5   picked6   picked7
##    -0.005     0.009     0.004    -0.002     0.019     0.023    -0.001    -0.001
##   picked8   picked9  picked10  picked11  picked12  picked13  picked14  picked15
##    -0.005     0.002    -0.001     0.010     0.000     0.026    -0.009     0.017
##  picked16  picked17  picked18  picked19  picked20  picked21  picked22  picked23
##    -0.002    -0.006    -0.001    -0.001    -0.002    -0.002     0.000     0.019
##  picked24  picked25  picked26  picked27  picked28  picked29  picked30  picked31
##    -0.002     0.013    -0.008     0.006     0.020    -0.002    -0.006     0.016
##  picked32  picked33  picked34  picked35  picked36  picked37  picked38  picked39
##    -0.004    -0.003    -0.005    -0.003     0.016    -0.001    -0.001     0.000
##  picked40  picked41  picked42  picked43  picked44  picked45  picked46  picked47
##    -0.001     0.000    -0.002    -0.006    -0.001    -0.001     0.001    -0.001
##  picked48  picked49  picked50  picked51  picked52  picked53  picked54  picked55
##     0.003     0.031    -0.005    -0.002     0.025     0.000     0.017     0.000
##  picked56  picked57  picked58  picked59  picked60  picked61  picked62  picked63
##    -0.002     0.007    -0.002    -0.002    -0.001    -0.001    -0.001    -0.004
##  picked64  picked65  picked66  picked67  picked68  picked69  picked70  picked71
##    -0.008    -0.001    -0.001     0.000    -0.005    -0.002     0.001     0.000
##  picked72  picked73  picked74  picked75  picked76  picked77  picked78  picked79
##    -0.001    -0.001     0.003     0.007    -0.001    -0.002    -0.004     0.000
```

```
##  picked80  picked81  picked82  picked83  picked84  picked85  picked86  picked87
##    -0.001    -0.005    -0.002     0.000     0.032    -0.001    -0.001    -0.001
##  picked88  picked89  picked90  picked91  picked92  picked93  picked94  picked95
##    -0.001    -0.006    -0.003     0.000     0.000    -0.003    -0.001    -0.007
##  picked96  picked97  picked98  picked99 picked100 picked101 picked102 picked103
##     0.026    -0.005     0.019     0.013    -0.003     0.005    -0.001     0.000
## picked104 picked105 picked106 picked107 picked108 picked109 picked110 picked111
##     0.016    -0.001     0.022    -0.003    -0.003    -0.001    -0.002    -0.004
## picked112 picked113 picked114 picked115 picked116 picked117 picked118 picked119
##    -0.001     0.000    -0.002    -0.001    -0.001     0.000    -0.008    -0.005
## picked120 picked121 picked122 picked123 picked124 picked125 picked126 picked127
##     0.009    -0.002     0.000    -0.001    -0.002    -0.001    -0.005    -0.004
## picked128 picked129 picked130 picked131 picked132 picked133 picked134 picked135
##    -0.001    -0.001    -0.001    -0.001    -0.001     0.000    -0.001    -0.001
## picked136 picked137 picked138 picked139 picked140 picked141 picked142 picked143
##     0.000    -0.006    -0.005    -0.001    -0.001    -0.001    -0.001    -0.001
## picked144 picked145 picked146 picked147 picked148 picked149 picked150 picked151
##    -0.001    -0.001    -0.001    -0.001    -0.001    -0.001    -0.001     0.004
## picked152 picked153 picked154 picked155 picked156 picked157 picked158 picked159
##    -0.001    -0.001    -0.001    -0.001    -0.007    -0.001    -0.008    -0.001
## picked160 picked161 picked162 picked163 picked164 picked165 picked166 picked167
##    -0.002    -0.001    -0.001     0.062    -0.001    -0.002     0.004    -0.001
## picked168 picked169 picked170 picked171 picked172 picked173 picked174 picked175
##    -0.001    -0.001    -0.005    -0.001     0.002    -0.007     0.017     0.050
## picked176 picked177 picked178 picked179 picked180 picked181 picked182 picked183
##    -0.002     0.028    -0.001    -0.007     0.016     0.013    -0.002    -0.001
## picked184 picked185 picked186 picked187 picked188 picked189 picked190 picked191
##    -0.001    -0.003    -0.001    -0.001    -0.007    -0.001    -0.005     0.006
## picked192 picked193 picked194 picked195 picked196 picked197 picked198 picked199
##    -0.001    -0.001    -0.001     0.000    -0.007    -0.004     0.008    -0.001
## picked200 picked201 picked202 picked203 picked204 picked205 picked206 picked207
##    -0.002    -0.001    -0.003    -0.001    -0.005    -0.002    -0.002    -0.001
## picked208 picked209
##    -0.001     0.000
```

```
# write DW calibrated to GBD to Excel
openxlsx::write.xlsx(cbind(DW_table[, c("names",
                                        "DW_GBD_All",
                                        "CI_lower_GBD_All",
                                        "CI_upper_GBD_All")],
                           paste0("[", sprintf("%.3f",
                                               DW_table[,"CI_lower_GBD_All"]),
                                  "; ", sprintf("%.3f",
                                                DW_table[,"CI_upper_GBD_All"]),
                                  "]")),
                     rowNames=FALSE,
                     file=paste0(file.path, "/Results/DW_GBD.xlsx"))
```

Task 3: Spearman correlation coefficients:demographic attributes

```r
# define levels of variables
education.names <- c("LowEducation", "MiddleEducation", "HighEducation")
data$opleiding_cat <- factor(x=data$opleiding_cat, levels = 1:3,
                             labels = education.names)

sex.names <- c("Male", "Female")
data$geslacht <- factor(x=data$geslacht, levels = 1:2, labels = sex.names)

age.names <- c("18-34yrs.", "35-54yrs.", "55-75yrs.")
data$leeftijd_3cat <- factor(x=data$leeftijd_3cat, levels = 1:3,
                             labels = age.names)

disease_exp.names <- c("No chronic disease", "Yes chronic disease")
data$disease_exp <- factor(x=data$disease_exp, levels = 0:1,
                           labels = disease_exp.names)

region.names <- c("North", "East", "South", "West")
data$regio_4cat <- factor(x=data$regio_4cat, levels = 1:4,
                          labels = region.names)

# probit regression for different strata
for (education in education.names){
  data_education <- data[data$opleiding_cat==education,]
  assign(paste0("data_", education), data_education)

  output_education <- stats::glm(formula, family=binomial(link="probit"),
                                 data=data_education)
  assign(paste0("output_", education), output_education)
  assign(paste0("coef_", education),
         summary(output_education)$coefficients[,1])
}

for (sex in sex.names){
  data_sex <- data[data$geslacht==sex,]
  assign(paste0("data_", sex), data_sex)

  output_sex <- stats::glm(formula, family=binomial(link="probit"),
                           data=data_sex)
  assign(paste0("output_", sex), output_sex)
  assign(paste0("coef_", sex), summary(output_sex)$coefficients[,1])
}

for (age in age.names){
  data_age <- data[data$leeftijd_3cat==age,]
  assign(paste0("data_", age), data_age)

  output_age <- stats::glm(formula, family=binomial(link="probit"),
                           data=data_age)
  assign(paste0("output_", age), output_age)
  assign(paste0("coef_", age), summary(output_age)$coefficients[,1])
}
```

```r
for (disease_exp in disease_exp.names){
  data_disease_exp <- data[data$disease_exp==disease_exp,]
  assign(paste0("data_", disease_exp), data_disease_exp)

  output_disease_exp <- stats::glm(formula, family=binomial(link="probit"),
                                   data=data_disease_exp)
  assign(paste0("output_", disease_exp), output_disease_exp)
  assign(paste0("coef_", disease_exp),
         summary(output_disease_exp)$coefficients[,1])
}

for (region in region.names){
  data_region <- data[data$regio_4cat==region,]
  assign(paste0("data_", region), data_region)

  output_region <- stats::glm(formula, family=binomial(link="probit"),
                              data=data_region)
  assign(paste0("output_", region), output_region)
  assign(paste0("coef_", region), summary(output_region)$coefficients[,1])
}

# define plot minimum and maximum
plot.min <- min(get("coef_LowEducation"),
                get("coef_MiddleEducation"),
                get("coef_HighEducation"),
                get("coef_All"),
                get("coef_Male"),
                get("coef_Female"),
                get("coef_18-34yrs."),
                get("coef_35-54yrs."),
                get("coef_55-75yrs."),
                get("coef_No chronic disease"),
                get("coef_Yes chronic disease"),
                get("coef_North"),
                get("coef_East"),
                get("coef_South"),
                get("coef_West"))
plot.max <- max(get("coef_LowEducation"),
                get("coef_MiddleEducation"),
                get("coef_HighEducation"),
                get("coef_All"),
                get("coef_Male"),
                get("coef_Female"),
                get("coef_18-34yrs."),
                get("coef_35-54yrs."),
                get("coef_55-75yrs."),
                get("coef_No chronic disease"),
                get("coef_Yes chronic disease"),
                get("coef_North"),
                get("coef_East"),
                get("coef_South"),
                get("coef_West"))
if (abs(plot.min) < plot.max){
```

```
  plot.min <- -plot.max
} else{
  plot.max <- abs(plot.min)
}

# make plots with reference low education
coef_All <- coef_All[-nr.health.states]

# plot stratified by education
png(file=paste0(file.path, "/Results/Coefficients_by_education.png"),
    width=32, height=32, units="cm", res=300)
par(mfrow=c(3, 3))

plot(x=coef_LowEducation, y=coef_All,
     xlab="Low education", ylab="All",
     xlim=c(plot.min, plot.max), ylim=c(plot.min, plot.max),
     col="#2596be", cex.lab=1.5, frame.plot=TRUE)
text(x=plot.min+0.2, y=plot.max-0.1,
     labels=paste0("r=", sprintf("%.3f", cor(coef_LowEducation,
                                             coef_All,
                                             method="spearman"))))
abline(a=0, b=1, col="#AD002AFF", lty=2)

plot(x=coef_LowEducation, y=coef_MiddleEducation,
     xlab="Low education", ylab="Middle education",
     xlim=c(plot.min, plot.max), ylim=c(plot.min, plot.max),
     col="#2596be", cex.lab=1.5, frame.plot=TRUE)
text(x=plot.min+0.2, y=plot.max-0.1,
     labels=paste0("r=", sprintf("%.3f", cor(coef_LowEducation,
                                             coef_MiddleEducation,
                                             method="spearman"))))
abline(a=0, b=1, col="#AD002AFF", lty=2)

plot(x=coef_LowEducation, y=coef_HighEducation,
     xlab="Low education", ylab="High education",
     xlim=c(plot.min, plot.max), ylim=c(plot.min, plot.max),
     col="#2596be", cex.lab=1.5, frame.plot=TRUE)
text(x=plot.min+0.2, y=plot.max-0.1,
     labels=paste0("r=", sprintf("%.3f", cor(coef_LowEducation,
                                             coef_HighEducation,
                                             method="spearman"))))
abline(a=0, b=1, col="#AD002AFF", lty=2)

# make plots with reference middle education
plot(x=coef_MiddleEducation, y=coef_All,
     xlab="Middle education", ylab="All",
     xlim=c(plot.min, plot.max), ylim=c(plot.min, plot.max),
     col="#2596be", cex.lab=1.5, frame.plot=TRUE)
text(x=plot.min+0.2, y=plot.max-0.1,
     labels=paste0("r=", sprintf("%.3f", cor(coef_MiddleEducation,
                                             coef_All,
                                             method="spearman"))))
abline(a=0, b=1, col="#AD002AFF", lty=2)
```

```
plot(x=coef_MiddleEducation, y=coef_HighEducation,
     xlab="Middle education", ylab="High education",
     xlim=c(plot.min, plot.max), ylim=c(plot.min, plot.max),
     col="#2596be", cex.lab=1.5, frame.plot=TRUE)
text(x=plot.min+0.2, y=plot.max-0.1,
     labels=paste0("r=", sprintf("%.3f", cor(coef_MiddleEducation,
                                             coef_HighEducation,
                                             method="spearman"))))
abline(a=0, b=1, col="#AD002AFF", lty=2)
plot.new()

# make plots with reference high education
plot(x=coef_HighEducation, y=coef_All,
     xlab="High education", ylab="All",
     xlim=c(plot.min, plot.max), ylim=c(plot.min, plot.max),
     col="#2596be", cex.lab=1.5, frame.plot=TRUE)
text(x=plot.min+0.2, y=plot.max-0.1,
     labels=paste0("r=", sprintf("%.3f", cor(coef_HighEducation,
                                             coef_All,
                                             method="spearman"))))
abline(a=0, b=1, col="#AD002AFF", lty=2)
plot.new()
plot.new()
grDevices::dev.off()
```

```
## pdf
##   2
```

```
# plot stratified by sex
png(file=paste0(file.path, "/Results/Coefficients_by_sex.png"),
    width=32, height=32, units="cm", res=300)
par(mfrow=c(2, 2))

# make plots with reference males
plot(x=coef_Male, y=coef_All,
     xlab="Male", ylab="All",
     xlim=c(plot.min, plot.max), ylim=c(plot.min, plot.max),
     col="#2596be", cex.lab=1.5, frame.plot=TRUE)
text(x=plot.min+0.2, y=plot.max-0.1,
     labels=paste0("r=", sprintf("%.3f", cor(coef_Male,
                                             coef_All,
                                             method="spearman"))))
abline(a=0, b=1, col="#AD002AFF", lty=2)

plot(x=coef_Male, y=coef_Female,
     xlab="Male", ylab="Female",
     xlim=c(plot.min, plot.max), ylim=c(plot.min, plot.max),
     col="#2596be", cex.lab=1.5, frame.plot=TRUE)
text(x=plot.min+0.2, y=plot.max-0.1,
     labels=paste0("r=", sprintf("%.3f", cor(coef_Male,
                                             coef_Female,
                                             method="spearman"))))
abline(a=0, b=1, col="#AD002AFF", lty=2)
```

```r
# make plots with reference females
plot(x=coef_Female, y=coef_All,
     xlab="Female", ylab="All",
     xlim=c(plot.min, plot.max), ylim=c(plot.min, plot.max),
     col="#2596be", cex.lab=1.5, frame.plot=TRUE)
text(x=plot.min+0.2, y=plot.max-0.1,
     labels=paste0("r=", sprintf("%.3f", cor(coef_Female,
                                             coef_All,
                                             method="spearman"))))
abline(a=0, b=1, col="#AD002AFF", lty=2)
plot.new()
grDevices::dev.off()
```

```
## pdf
##   2
```

```r
# plot stratified by age
png(file=paste0(file.path, "/Results/Coefficients_by_age.png"),
    width=32, height=32, units="cm", res=300)
par(mfrow=c(3, 3))

# make plots with reference young individuals
plot(x=get("coef_18-34yrs."), y=coef_All,
     xlab="18-34 yrs.", ylab="All",
     xlim=c(plot.min, plot.max), ylim=c(plot.min, plot.max),
     col="#2596be", cex.lab=1.5, frame.plot=TRUE)
text(x=plot.min+0.2, y=plot.max-0.1,
     labels=paste0("r=", sprintf("%.3f", cor(get("coef_18-34yrs."),
                                             coef_All,
                                             method="spearman"))))
abline(a=0, b=1, col="#AD002AFF", lty=2)

plot(x=get("coef_18-34yrs."), y=get("coef_35-54yrs."),
     xlab="18-34 yrs.", ylab="35-54 yrs.",
     xlim=c(plot.min, plot.max), ylim=c(plot.min, plot.max),
     col="#2596be", cex.lab=1.5, frame.plot=TRUE)
text(x=plot.min+0.2, y=plot.max-0.1,
     labels=paste0("r=", sprintf("%.3f", cor(get("coef_18-34yrs."),
                                             get("coef_35-54yrs."),
                                             method="spearman"))))
abline(a=0, b=1, col="#AD002AFF", lty=2)

plot(x=get("coef_18-34yrs."), y=get("coef_55-75yrs."),
     xlab="18-34 yrs.", ylab="55-75 yrs.",
     xlim=c(plot.min, plot.max), ylim=c(plot.min, plot.max),
     col="#2596be", cex.lab=1.5, frame.plot=TRUE)
text(x=plot.min+0.2, y=plot.max-0.1,
     labels=paste0("r=", sprintf("%.3f", cor(get("coef_18-34yrs."),
                                             get("coef_55-75yrs."),
                                             method="spearman"))))
abline(a=0, b=1, col="#AD002AFF", lty=2)
```

```r
# make plots with reference middle-aged individuals
plot(x=get("coef_35-54yrs."), y=coef_All,
     xlab="35-54 yrs.", ylab="All",
     xlim=c(plot.min, plot.max), ylim=c(plot.min, plot.max),
     col="#2596be", cex.lab=1.5, frame.plot=TRUE)
text(x=plot.min+0.2, y=plot.max-0.1,
     labels=paste0("r=", sprintf("%.3f", cor(get("coef_35-54yrs."),
                                             coef_All, method="spearman"))))
abline(a=0, b=1, col="#AD002AFF", lty=2)

plot(x=get("coef_35-54yrs."), y=get("coef_55-75yrs."),
     xlab="35-54 yrs.", ylab="55-75 yrs.",
     xlim=c(plot.min, plot.max), ylim=c(plot.min, plot.max),
     col="#2596be", cex.lab=1.5, frame.plot=TRUE)
text(x=plot.min+0.2, y=plot.max-0.1,
     labels=paste0("r=", sprintf("%.3f", cor(get("coef_35-54yrs."),
                                             get("coef_55-75yrs."),
                                             method="spearman"))))
abline(a=0, b=1, col="#AD002AFF", lty=2)
plot.new()

# make plots with reference old individuals
plot(x=get("coef_55-75yrs."), y=coef_All,
     xlab="55-75 yrs.", ylab="All",
     xlim=c(plot.min, plot.max), ylim=c(plot.min, plot.max),
     col="#2596be", cex.lab=1.5, frame.plot=TRUE)
text(x=plot.min+0.2, y=plot.max-0.1,
     labels=paste0("r=", sprintf("%.3f", cor(get("coef_55-75yrs."),
                                             coef_All,
                                             method="spearman"))))
abline(a=0, b=1, col="#AD002AFF", lty=2)
plot.new()
plot.new()
grDevices::dev.off()
```

```
## pdf
##   2
```

```r
# plot stratified by chronic disease status
png(file=paste0(file.path, "/Results/Coefficients_by_disease_exp.png"),
    width=32, height=32, units="cm", res=300)
par(mfrow=c(2, 2))

# make plots with reference No_chronic_disease
plot(x=get("coef_No chronic disease"), y=coef_All,
     xlab="No chronic disease", ylab="All",
     xlim=c(plot.min, plot.max), ylim=c(plot.min, plot.max),
     col="#2596be", cex.lab=1.5, frame.plot=TRUE)
text(x=plot.min+0.2, y=plot.max-0.1,
     labels=paste0("r=", sprintf("%.3f", cor(get("coef_No chronic disease"),
                                             coef_All, method="spearman"))))
abline(a=0, b=1, col="#AD002AFF", lty=2)
```

```r
plot(x=get("coef_No chronic disease"), y=get("coef_Yes chronic disease"),
     xlab="No chronic disease", ylab="Yes chronic disease",
     xlim=c(plot.min, plot.max), ylim=c(plot.min, plot.max),
     col="#2596be", cex.lab=1.5, frame.plot=TRUE)
text(x=plot.min+0.2, y=plot.max-0.1,
     labels=paste0("r=", sprintf("%.3f", cor(get("coef_No chronic disease"),
                                             get("coef_Yes chronic disease"),
                                             method="spearman"))))
abline(a=0, b=1, col="#AD002AFF", lty=2)

# make plots with reference Yes_chronic_disease
plot(x=get("coef_Yes chronic disease"), y=coef_All,
     xlab="Yes chronic disease", ylab="All",
     xlim=c(plot.min, plot.max), ylim=c(plot.min, plot.max),
     col="#2596be", cex.lab=1.5, frame.plot=TRUE)
text(x=plot.min+0.2, y=plot.max-0.1,
     labels=paste0("r=", sprintf("%.3f", cor(get("coef_Yes chronic disease"),
                                             coef_All, method="spearman"))))
abline(a=0, b=1, col="#AD002AFF", lty=2)
plot.new()
grDevices::dev.off()
```

```
## pdf
##   2
```

```r
# plot stratified by region
png(file=paste0(file.path, "/Results/Coefficients_by_region.png"),
    width=32, height=32, units="cm", res=300)
par(mfrow=c(4, 4))

# make plots with reference North
plot(x=get("coef_North"), y=coef_All,
     xlab="North", ylab="All",
     xlim=c(plot.min, plot.max), ylim=c(plot.min, plot.max),
     col="#2596be", cex.lab=1.5, frame.plot=TRUE)
text(x=plot.min+0.2, y=plot.max-0.1,
     labels=paste0("r=", sprintf("%.3f", cor(get("coef_North"),
                                             coef_All,
                                             method="spearman"))))
abline(a=0, b=1, col="#AD002AFF", lty=2)

plot(x=get("coef_North"), y=get("coef_East"),
     xlab="North", ylab="East",
     xlim=c(plot.min, plot.max), ylim=c(plot.min, plot.max),
     col="#2596be", cex.lab=1.5, frame.plot=TRUE)
text(x=plot.min+0.2, y=plot.max-0.1,
     labels=paste0("r=", sprintf("%.3f", cor(get("coef_North"),
                                             get("coef_East"),
                                             method="spearman"))))
abline(a=0, b=1, col="#AD002AFF", lty=2)
```

```r
plot(x=get("coef_North"), y=get("coef_South"),
     xlab="North", ylab="South",
     xlim=c(plot.min, plot.max), ylim=c(plot.min, plot.max),
     col="#2596be", cex.lab=1.5, frame.plot=TRUE)
text(x=plot.min+0.2, y=plot.max-0.1,
     labels=paste0("r=", sprintf("%.3f", cor(get("coef_North"),
                                             get("coef_South"),
                                             method="spearman"))))
abline(a=0, b=1, col="#AD002AFF", lty=2)

plot(x=get("coef_North"), y=get("coef_West"),
     xlab="North", ylab="West",
     xlim=c(plot.min, plot.max), ylim=c(plot.min, plot.max),
     col="#2596be", cex.lab=1.5, frame.plot=TRUE)
text(x=plot.min+0.2, y=plot.max-0.1,
     labels=paste0("r=", sprintf("%.3f", cor(get("coef_North"),
                                             get("coef_West"),
                                             method="spearman"))))
abline(a=0, b=1, col="#AD002AFF", lty=2)

# make plots with reference East
plot(x=get("coef_East"), y=coef_All,
     xlab="East", ylab="All",
     xlim=c(plot.min, plot.max), ylim=c(plot.min, plot.max),
     col="#2596be", cex.lab=1.5, frame.plot=TRUE)
text(x=plot.min+0.2, y=plot.max-0.1,
     labels=paste0("r=", sprintf("%.3f", cor(get("coef_East"),
                                             coef_All,
                                             method="spearman"))))
abline(a=0, b=1, col="#AD002AFF", lty=2)

plot(x=get("coef_East"), y=get("coef_West"),
     xlab="East", ylab="West",
     xlim=c(plot.min, plot.max), ylim=c(plot.min, plot.max),
     col="#2596be", cex.lab=1.5, frame.plot=TRUE)
text(x=plot.min+0.2, y=plot.max-0.1,
     labels=paste0("r=", sprintf("%.3f", cor(get("coef_East"),
                                             get("coef_West"),
                                             method="spearman"))))
abline(a=0, b=1, col="#AD002AFF", lty=2)

plot(x=get("coef_East"), y=get("coef_South"),
     xlab="East", ylab="South",
     xlim=c(plot.min, plot.max), ylim=c(plot.min, plot.max),
     col="#2596be", cex.lab=1.5, frame.plot=TRUE)
text(x=plot.min+0.2, y=plot.max-0.1,
     labels=paste0("r=", sprintf("%.3f", cor(get("coef_East"),
                                             get("coef_South"),
                                             method="spearman"))))
abline(a=0, b=1, col="#AD002AFF", lty=2)

# make plots with reference South
plot(x=get("coef_South"), y=coef_All,
```

```
     xlab="South", ylab="All",
     xlim=c(plot.min, plot.max), ylim=c(plot.min, plot.max),
     col="#2596be", cex.lab=1.5, frame.plot=TRUE)
text(x=plot.min+0.2, y=plot.max-0.1,
     labels=paste0("r=", sprintf("%.3f", cor(get("coef_South"),
                                             coef_All,
                                             method="spearman"))))
abline(a=0, b=1, col="#AD002AFF", lty=2)

plot(x=get("coef_South"), y=get("coef_West"),
     xlab="South", ylab="West",
     xlim=c(plot.min, plot.max), ylim=c(plot.min, plot.max),
     col="#2596be", cex.lab=1.5, frame.plot=TRUE)
text(x=plot.min+0.2, y=plot.max-0.1,
     labels=paste0("r=", sprintf("%.3f", cor(get("coef_South"),
                                             get("coef_West"),
                                             method="spearman"))))
abline(a=0, b=1, col="#AD002AFF", lty=2)

# make plots with reference West
plot(x=get("coef_West"), y=coef_All,
     xlab="West", ylab="All",
     xlim=c(plot.min, plot.max), ylim=c(plot.min, plot.max),
     col="#2596be", cex.lab=1.5, frame.plot=TRUE)
text(x=plot.min+0.2, y=plot.max-0.1,
     labels=paste0("r=", sprintf("%.3f", cor(get("coef_West"),
                                             coef_All,
                                             method="spearman"))))
abline(a=0, b=1, col="#AD002AFF", lty=2)
grDevices::dev.off()
```

```
## pdf
##   2
```

```
# combine stratified figures
png(file=paste0(file.path, "/Results/Coefficients_Stratified.png"),
    width=40, height=10, units="cm", res=300)
par(mar=rep(0, 4))
layout(matrix(1:5, ncol=5, byrow=FALSE))
for (name in c("education", "age", "sex", "disease_exp", "region")){
  plot(NA, xlim=0:1, ylim=0:1, xaxt="n", yaxt="n", bty="n")
  img <- png::readPNG(paste0(file.path, "/Results/Coefficients_by_",
                             name, ".png"))
  rasterImage(img, 0, 0, 1, 1)
}
if (dev.cur() >1){
  dev.off()
}
```

```
## pdf
##   2
```

```
knitr::include_graphics(paste0(file.path, "/Results/Coefficients_by_education.png"))
```

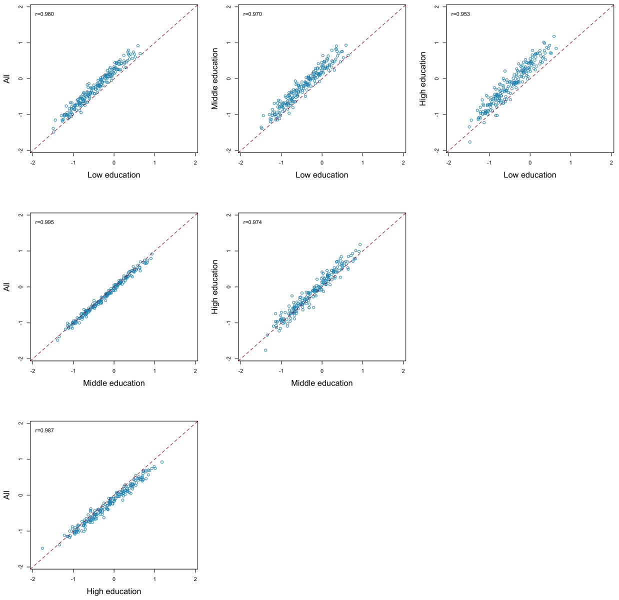


```
knitr::include_graphics(paste0(file.path, "/Results/Coefficients_by_sex.png"))
```

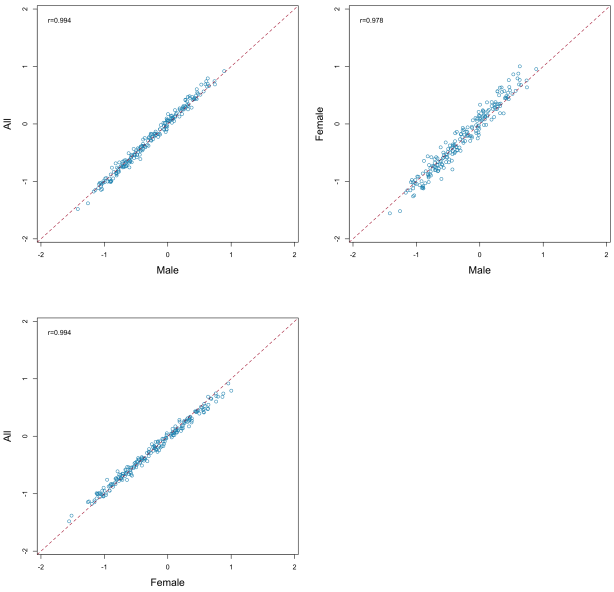


```
knitr::include_graphics(paste0(file.path, "/Results/Coefficients_by_age.png"))
```

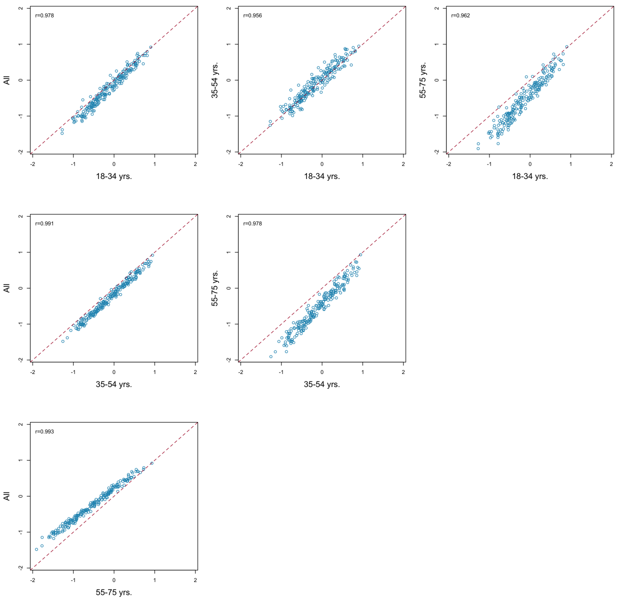


```r
knitr::include_graphics(paste0(file.path, "/Results/Coefficients_by_disease_exp.png"))
```

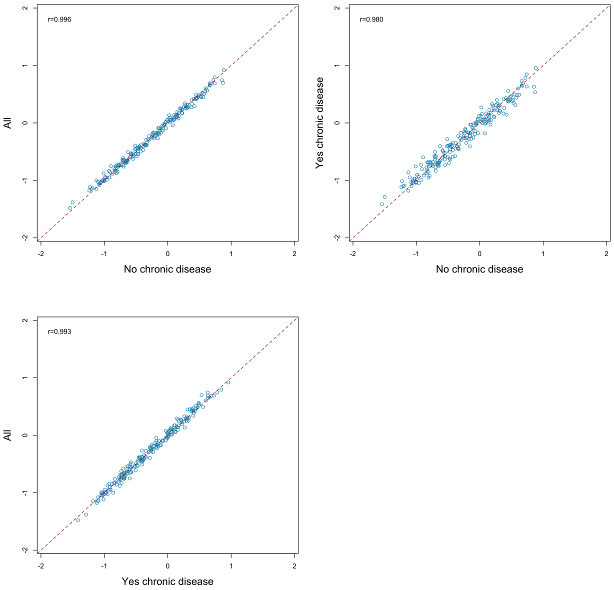


```r
knitr::include_graphics(paste0(file.path, "/Results/Coefficients_by_region.png"))
```

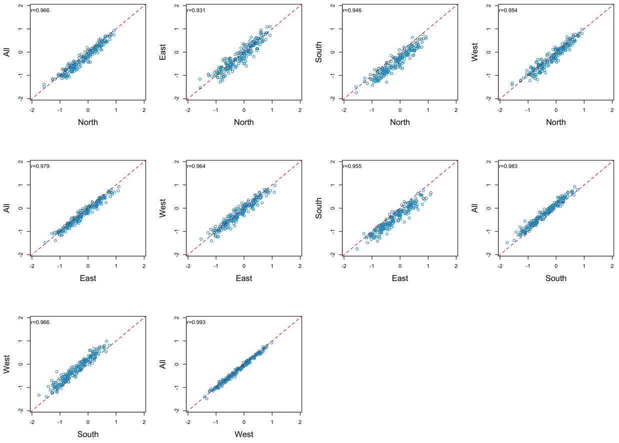
r=0.966
All
North
r=0.931
East
North
r=0.946
South
North
r=0.954
West
North
r=0.979
All
East
r=0.964
West
East
r=0.955
South
East
r=0.983
All
South
r=0.966
West
South
r=0.993
All
West
